# The effect of radiofrequency electromagnetic fields (RF-EMF) on biomarkers of oxidative stress *in vivo* and *in vitro*: A protocol for a systematic review

**DOI:** 10.1016/j.envint.2021.106932

**Published:** 2022-01

**Authors:** Bernd Henschenmacher, Annette Bitsch, Tonia de las Heras Gala, Henry Jay Forman, Athanassios Fragoulis, Pietro Ghezzi, Rupert Kellner, Wolfgang Koch, Jens Kuhne, Dmitrij Sachno, Gernot Schmid, Katya Tsaioun, Jos Verbeek, Robert Wright

**Affiliations:** a Federal Office for Radiation Protection, Ingolstädter Landstraße 1, 85764 Oberschleißheim, Germany; b Fraunhofer Institute for Toxicology and Experimental Medicine, Chemical Safety and Toxicology, Nikolai-Fuchs-Straße 1, 30625 Hannover, Germany; c Leonard Davis School of Gerontology, University of Southern California, 3715 McClintock Avenue, Los Angeles, CA 90089, USA; d University of California Merced, 5200 Lake Road, Merced, CA 95343, USA; e Department of Anatomy and Cell Biology, Uniklinik RWTH Aachen, Wendlingweg 2, 52074 Aachen, Germany; f Brighton and Sussex Medical School, University of Sussex, Trafford Centre, Falmer BN1 9RY, United Kingdom; g Department of Biomolecular Sciences, University of Urbino Carlo Bo, Urbino, Italy; h Seibersdorf Laboratories, Campus Seibersdorf, 2444 Seibersdorf, Austria; i Evidence-based Toxicology Collaboration (EBTC), Johns Hopkins Bloomberg School of Public Health, Baltimore, MD 21205, USA; j University Medical Center Amsterdam, Cochrane Work, Meibergdreef 9, 1105 AZ Amsterdam, the Netherlands; k William H. Welch Medical Library, Johns Hopkins University School of Medicine, 2024 E. Monument Street, Suite 1-200, Baltimore, MD 21205, USA

**Keywords:** SAR, W/kg, Oxidative stress, Radiofrequency electromagnetic fields, High frequency electromagnetic fields, ROS, Free radicals, Electrophilic species, Systematic review

## Abstract

**Background:**

Oxidative stress is conjectured to be related to many diseases. Furthermore, it is hypothesized that radiofrequency fields may induce oxidative stress in various cell types and thereby compromise human and animal health. This systematic review (SR) aims to summarize and evaluate the literature related to this hypothesis.

**Objectives:**

The main objective of this SR is to evaluate the associations between the exposure to radiofrequency electromagnetic fields and oxidative stress in experimental models (*in vivo* and *in vitro*).

**Methods:**

The SR framework has been developed following the guidelines established in the *WHO Handbook for Guideline Development* and the *Handbook for Conducting a Literature-Based Health Assessment).* We will include controlled *in vivo* and *in vitro* laboratory studies that assess the effects of an exposure to RF-EMF on valid markers for oxidative stress compared to no or sham exposure. The protocol is registered in PROSPERO.

We will search the following databases: PubMed, Embase, Web of Science Core Collection, Scopus, and the EMF-Portal. The reference lists of included studies and retrieved review articles will also be manually searched.

**Study appraisal and synthesis method:**

Data will be extracted according to a pre-defined set of forms developed in the DistillerSR online software and synthesized in a *meta*-analysis when studies are judged sufficiently similar to be combined. If a *meta*-analysis is not possible, we will describe the effects of the exposure in a narrative way.

**Risk of bias:**

The risk of bias will be assessed with the NTP/OHAT risk of bias rating tool for human and animal studies.

We will use GRADE to assess the certainty of the conclusions (high, moderate, low, or inadequate) regarding the association between radiofrequency electromagnetic fields and oxidative stress.

**Funding:**

This work was funded by the World Health Organization (WHO).

**Registration:**

The protocol was registered on the PROSPERO webpage on July 8, 2021.

## Graphical abstract

**Figure:**
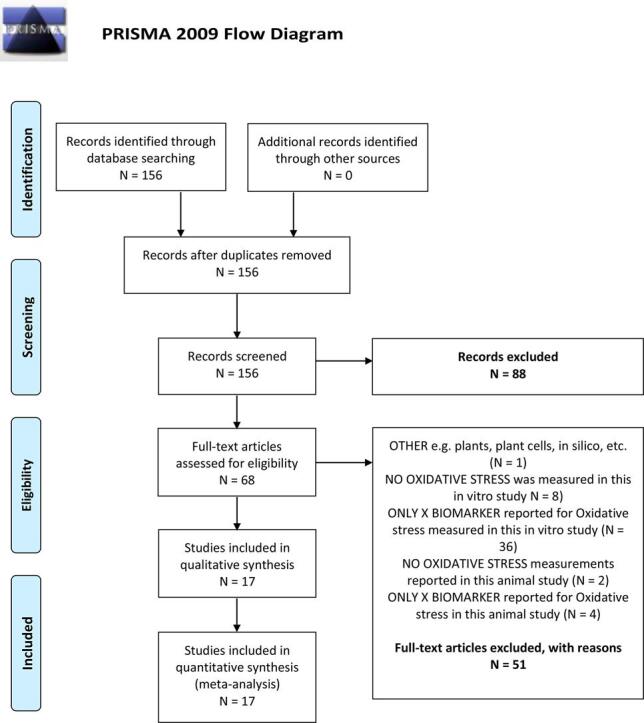

## 1 Background

The technological applications of radiofrequency electromagnetic fields (RF-EMF) have been steadily increasing since the 1950s. RF-EMF are used in medicine^1^ (e.g. magnetic resonance imaging, diathermy, radiofrequency ablation); industry (e.g. heating and welding); domestic appliances (e.g. baby monitors, Wi-Fi); security and navigation (e.g. radar and RFID); and especially in telecommunications (e.g. radio and TV broadcasting, mobile telephony). These developments mean that large parts of the global population are now exposed to RF-EMF and more will be exposed in the future. Concern has been raised regarding public health consequences from RF-EMF, and it is therefore crucial to perform a health risk assessment to support decision-makers and inform the general public.

The World Health Organization (WHO) has an ongoing project to assess potential health effects of exposure to RF-EMF in the general and working population. To prioritize potential adverse health outcomes from exposure to these fields, WHO conducted a broad international survey amongst RF experts in 2018 (Verbeek et al., 2021). Six major topics were identified (cancer, adverse reproductive outcomes, cognitive impairment, electromagnetic hypersensitivity, oxidative stress, and heat related effects) for which WHO has commissioned systematic reviews of observational and experimental studies to analyze and synthesize the available evidence. In the current paper, we present the protocol for a systematic review of experimental studies on exposure to RF fields and biomarkers of oxidative stress.

### 1.1 Description of the outcome

Partially reduced species derived from oxygen, most of which can act as oxidants, are generated during a variety of biochemical reactions and cellular functions. These are often referred to erroneously as either free radicals, which only some are, or reactive oxygen species (or ROS). ROS is unfortunately too often used as if it were a molecular entity rather than an abbreviation for a group of species with markedly different chemistries. While hydroxyl radical can oxidize any organic molecule, hydrogen peroxide (H_2_O_2_) is the dominant physiological redox signaling molecule and has limited ability to oxidize organic molecules without metal or enzymatic catalysis (Forman et al., 2014).

Exogenous factors can also cause the generation of oxidants, for example, alcohol, cigarette smoke, environmental pollutants, and ionizing radiation (Møller et al., 1996). Oxidative stress occurs when the production of oxidants overrides the antioxidant capability of the target cell (Sies, 1985). Oxidants, particularly in the presence of metals, react with several cellular components, including proteins, lipids, and DNA, resulting in altered cell functions (Sies, 1985).

Oxidative damage has been hypothesized to occur in aging and degenerative diseases such as cancer and cardiovascular disease, even though the causal relationships are not fully elucidated. Oxidative stress is a state or a mechanism that cannot be measured in a simple way, in part because oxidants have very short half-lives. Various biomarkers have been proposed to represent the state of oxidative stress, typically oxidation products of lipids, proteins, and nucleic acids (Frijhoff et al., 2015). Oxidative stress is not a health outcome per se (meaning here any influence on health), but it could provide evidence of a mechanism by which radiofrequency radiation exposure might affect health. This is also called mechanistic evidence. For the role of oxidative stress in the adverse health outcomes mentioned, there are often only observational epidemiological studies available.

Non-ionizing radiation will not directly produce radical species, but there are potential mechanisms within cells by which RF-EMF may cause oxidative stress. However, the association between exposure to radiofrequency fields and oxidative stress remains unclear. Therefore, our review of the effects of RF exposure on oxidative stress, independent of the potential biological consequences yet to be established, can provide important information to guide future research in the field.

### 1.2 Description of the exposure

In the context of radiation protection, RF-EMF are defined as electromagnetic fields with frequencies from 100 kHz − 300 GHz (ICNIRP, 2020). Such fields are generated by many types of equipment both in the general living environment and in workplaces. We will consider two types of RF-EMF exposure in our review – localized exposure, typically occurring when the source of RF-EMF is operated close to the body (e.g. as with mobile phones), and whole-body exposure, as occurs in the general environment. Whole-body exposure in the general population results typically from RF-EMF sources located at a distance from the body, for example, radio and television masts, mobile phone base stations, digital enhanced cordless telecommunications (DECT) base stations, wireless local area network (WLAN) technologies, baby monitors, smart meters, and ambient exposure from otherś mobile phones. These are typical exposure situations in everyday life.

Exposure to radiofrequency electric, magnetic, and electromagnetic fields between 100 kHz and 300 GHz results in the induction of *internal electric fields* (electric field strength, E, expressed in V/m) or the absorption of electromagnetic energy (typically described by the *specific absorption rate*, *SAR*, expressed as W/kg) within the tissue or sample (Durney et al, 1986). At frequencies above 6 GHz, the absorption only takes place in very superficial regions of the exposed object and the *transmitted power density*, *PD_tr_* expressed in *W/m^2^* can be used to describe exposure within the tissue (Foster et al., 2016, Funahashi et al., 2018). In some cases, particularly for short pulses, *specific absorption, SA*, expressed in *J/kg* (or *transmitted energy density ED_tr_* at f > 6 GHz expressed in J/m^2^), can also be seen as an appropriate tissue internal exposure metric (Kodera et al., 2018, Foster et al., 2016). The magnitude of these tissue internal exposure metrics may be related to biological responses that are relevant for this review. Depending on *SAR | PD_tr_* and exposure time (or alternatively *SA | ED_tr_*), energy absorption can result in temperature increase, heat flux, thermoregulatory responses, or changes in the energy balance of the exposed biological system. This is possible across the whole frequency range (ICNIRP, 2020).

At frequencies below and within beta dispersion (f < 10 MHz), there might be an additional significant voltage drop across cell membranes. Therefore, the *internal electric field* strength itself could also be associated with biological outcomes (Schwan, 1985). *SAR* and *internal electric field* strength are related to each other via the electric conductivity and the mass density of the respective tissue. Moreover, *SAR* is causally related to *SA* via exposure time.

The magnitude and distribution of these internal exposure metrics strongly depend on the frequency, polarization, field strength, and field distribution of the external fields, as well as on the structure of the exposed object and the distribution of its electric properties (conductivity and permittivity). Therefore, describing only external exposure metrics, such as external field strength or external power density, is not sufficient to describe RF-EMF exposure and to evaluate potential exposure–response^2^ relationships (ICNIRP, 1998)).

In the MHz range, the coupling of the magnetic field with electron spins (radical pair mechanism) is a feasible mechanism for the potential interaction of RF-EMF with biochemical reactions (Timmel and Hore, 1996). During an exposure to homogenous magnetic fields (e.g. Helmholtz-coil exposure) at lower frequencies, the magnetic field distribution is not markedly altered by the exposed tissue because the penetration depth is typically higher than the dimensions of the tissue. For this special exposure scenario, the external homogenous magnetic field strength might be a valid proxy for the internal magnetic field inside the tissue. However, this kind of exposure also induces internal electric fields or SAR which have to be evaluated for a complete exposure characterization.

### 1.3 Rationale for a systematic review

One of the concerns of RF-EMF exposure is that it might increase oxidative stress in humans and therefore might be linked to adverse effects, leading to, for example, cancer and reduced fertility. There are numerous studies implying a link between electromagnetic fields and oxidative stress, including but not limited to fields in the radiofrequency range. A recent narrative review (Schuermann and Mevissen, 2021, see also Mevissen and Schürmann, 2021) on low frequency magnetic fields, high frequency electromagnetic fields, and oxidative stress indicates that RF-EMF may induce oxidative stress and thereby cause or contribute to reduced fertility and the onset of neurodegenerative diseases in humans. Other researchers have raised concerns that fields in the frequency range of 5G may have detrimental health effects due to oxidative stress (Desai et al., 2009, Di Ciaula, 2018). There is no consensus about the existence, extent, and mechanisms of oxidative stress induced by RF-EMF (see e.g. Schuermann and Mevissen, 2021) or whether RF-EMF induces persistent oxidative stress leading to damage in cells and organs (Jeong et al., 2018).

In a survey of RF-EMF experts, oxidative stress was rated as an important outcome that should be reviewed systematically (Verbeek et al., 2021). Oxidative stress could also contribute to the association between the exposure to polluted air and increased mortality (Rao et al., 2018).

## 2 Objectives

This SR will assess the effects of exposure to RF-EMF on biomarkers of oxidative stress in experimental *in vivo* and *in vitro* studies given the following PECO question (Population, Exposure, Comparator, and Outcome)

• *What is the effect of exposure to RF-EMF (E) on the most important and best validated biomarkers for oxidative stress (O) compared to no-exposure, sham exposure, or temperature-controlled no-exposure (C) in animals, humans, or cells (P)?*

## 3 Methods

The systematic review will be carried out according to the recommendations for systematic reviews as reported in the *WHO Handbook for Guideline Development* (World Health Organization, 2014) and the Handbook *for Conducting a Literature-Based Health Assessment* (NTP/OHAT, 2015) (see also Whaley et al., 2020).

The systematic review will be reported in accordance with the Preferred Reporting Items for Systematic Reviews and Meta-Analyses (PRISMA) guidelines (Moher et al., 2009).

The review team has expertise in systematic review methodology, redox processes in biology, oxidative stress markers, RF-EMF experimentation, and RF-EMF exposure assessment.

The review process will be managed using the DistillerSR web application. In case any methodological changes from this protocol are made during the review, they will be listed under the heading “Differences between protocol and review” in the final article. Disagreements between reviewers will be resolved by discussion; if no consensus can be reached a third reviewer will be involved.

### 3.1 Eligibility criteria

Only peer-reviewed research articles reporting original data will be included. Studies need to inform the elements of the PECO(S) (Population, Exposure, Comparators, Outcomes and Study Design) outlined below. All publication years will be considered, up to the search date. The references of reviews articles will be hand-searched.

#### 3.1.1 Populations

The review will include *in vivo* and *in vitro* experimental studies. *In vivo* models will include humans and mammals. *In vitro* studies will include:

• primary cells isolated from humans and mammals
• body fluids from humans and mammals
• immortalized and cancer cell lines, including differentiated cell lines of human and animal origin
• tissues and tissue fractions (humans and mammals)
• three-dimensional (3-D) culture models and organoids

We will exclude non-mammalian species as heat regulation differs markedly between mammals and other animals. Heat dissipation is important for the effects of RF-EMF and including animals with very different heat regulation mechanisms would make it difficult addressing our main research question.

#### 3.1.2 Types of exposures

(A) ***(body/tissue/sample) internal exposure metrics*** *measured or calculated for the particular conditions of the experiment*
  • *SAR* [*expressed in* W/kg *or equivalent units*]
  • *SA* [*expressed in* J/kg *or equivalent units*]
  • *induced electric field strength, [expressed in V/m or equivalent units]*
  • *internal magnetic field strength [expressed in A/m or equivalent units]*

(For exposures applied as pure or predominantly magnetic fields in the lower frequency range *the external magnetic field strength at the sample position* is considered a sufficient surrogate for the *tissue internal magnetic field*, as long as the penetration depth is high compared to the sample dimension)

(B) ***(body/tissue/sample) internal exposure metrics****describing superficial absorption at frequencies above 6 GHz measured or calculated for the conditions of the experiment as follows,*
  • *incident power flux density* [expressed in W/m^2^ or equivalent units]
  • *incident energy density* [expressed in J/m^2^ or equivalent units]
  • *transmitted (absorbed) power flux density* [expressed in W/m^2^ or equivalent units]
  • *transmitted (absorbed) energy density* [expressed in J/m^2^ or equivalent units],
(C) (body/tissue/sample) external exposure metrics
  • external electric field strength [V/m] (E > 1 V/m or E>√10*background level in unshielded environment, otherwise no restriction)
  • external magnetic field strength [mA/m] (H > 2.7 mA/m or H>√10*background level in unshielded environment, otherwise no restriction)
  • incident power flux density, [mW/m^2^] (PD > 2.5 mW/m^2^ or PD > 10*background level in unshielded environment, otherwise no restriction)

We will only include studies reporting external metrics under C if (i) either of these exposure metrics was measured or calculated at the location of the exposed body/tissue/sample in the approximate far-field of the field source, and (ii) the exposure level is at least a factor of 10 (power flux density) or √10 (field strength) above background level^3^. In the case where no specific background exposure level in the laboratory is reported in the study, we will assume a value of 0.25 mW/m^2^ (corresponding to 0.3 V/m and 0.9 mA/m, respectively) as the background exposure level. This results in an inclusion threshold of PD = 2.5 mW/m^2^, E = 1 V/m, or H = 2.7 mA/m).

(D) ***Mobile phones or other RF-generating devices as the source of exposure, without reporting of metrics under A, B, or C***

We will consider these studies separately because there is variation and/or uncertainty in exposure levels.

1. ***with output controlled by appropriate software or hardware operated close to the tissue/sample***

We will include studies that applied exposure with an output power controlled by hardware or software, provided that the output power and the distance to the sample are reported, thus enabling an inference of the exposure.

2. ***in GSM mode with an active call operated close to the body/tissue/sample***

An exposure applied as the field generated by a mobile phone in GSM mode with an active call operated at distances equal to or less than 3 cm from the body/tissue/sample can be expected to generate a temporal peak SAR in the range of 0.01–100 W/kg, at least a factor of 100 above the average background level. We will include these studies only if the active call was maintained throughout the experiment and the comparison was a similar phone switched off.

**Fig. 1:**
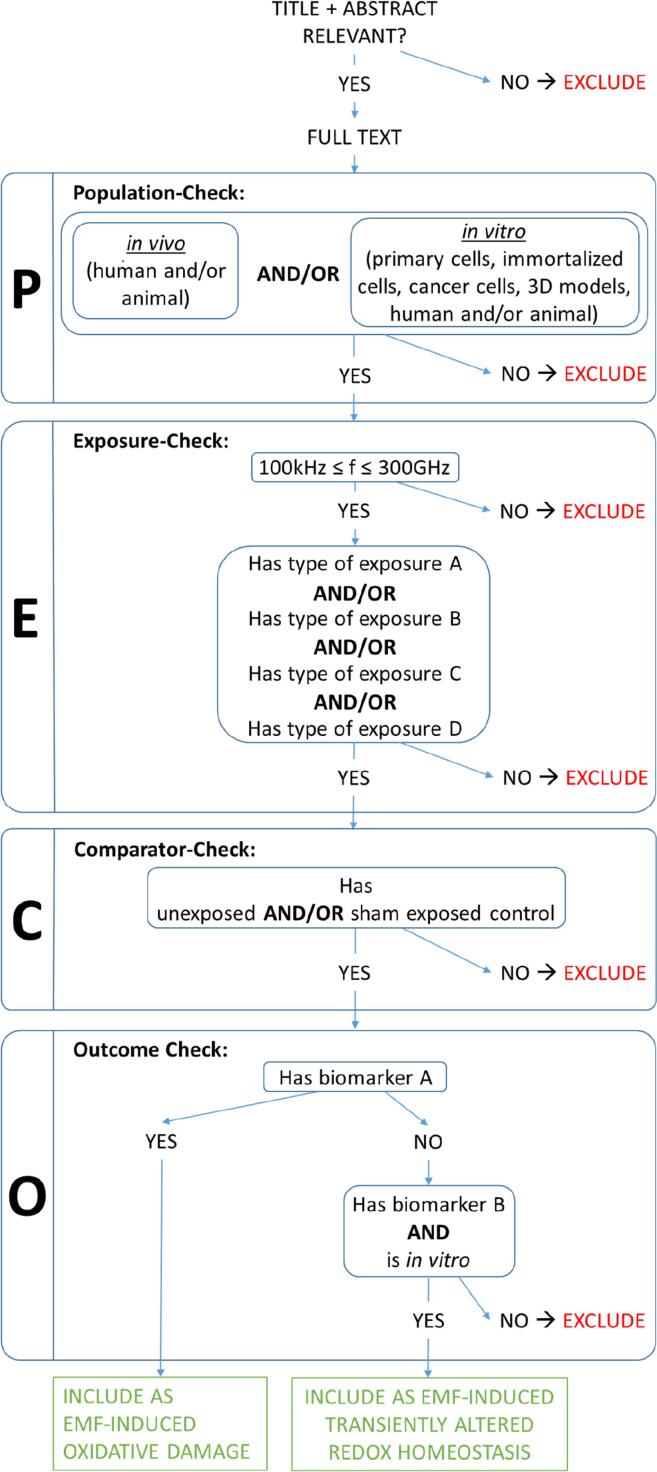
Decision tree for selecting studies based on exposure conditions and biomarkers of oxidative stress. EMF = electromagnetic field; E_ext_, H_ext_, B_ext_ = external electric field strength, magnetic field strength or magnetic flux density, respectively.

Fig. 1 presents how the exposure criteria will be used for the selection of studies.

#### 3.1.3 Comparators

We will only include studies that used at least one group exposed to RF-EMF and at least one separate reference group that is sham-exposed or not exposed to RF-EMF.

If the included studies used separate temperature control groups (i.e. in order to investigate the impact of exposure-induced heat generation), we will use this data as an additional comparator.

#### 3.1.4 Outcomes

The primary outcome of our systematic review will be the effects of RF-EMF on valid biomarkers of oxidative stress. Oxidative stress results from an imbalance between reactive chemical species and processes to reduce them. It is known to compromise the functions of cells, organs, and thereby organisms.

There is no commonly accepted single measure for oxidative stress. Various molecular biomarkers are considered to represent a state of oxidative stress. Commonly used biomarkers were evaluated with respect to their suitability as a valid measure of oxidative stress as discussed in the introduction.

All biomarkers were classified into either category “A” biomarkers (valid for *in vitro* and *in vivo* studies without limitations) or category “B” biomarkers (valid for *in vitro* studies, but only if measured as a real time course). Some biomarkers, even if used as indicators of “oxidative stress” in some studies, were considered non-specific and will not be assessed in the present review. Main examples of these are listed in Table 1 as category “X” biomarkers (not valid as measures of oxidative stress). We will only include studies that have examined at least one biomarker that matches the defined requirements to be classified as “A” (both for *in vivo* and *in vitro* studies) or “B” (for *in vitro* studies only), as we think only these are reliable indicators of oxidative processes within the cell.

**Table 1:** Markers of oxidative stress included in the systematic review.

Included markers of oxidative stress	Rating category	Comment
8-oxo-2′-deoxyguanosine (8-OHdG)	A	Damaged DNA difficult to do accurately
Chlorotyrosine	A	Requires inflammation
Glutathionylated protein/mixed protein disulfides	A	Reversibly modified proteins
4-hydroxy-2-nonenal/HNE/HNE-conjugated proteins	A	Lipid peroxidation
15F2-IsoProstane/8-*iso*-PGF2a	A	Common to all types of oxidative stress - must use additional prostaglandin to account for possible enzymatic production
Methionine sulfoxide and cysteic acid	A	Damaged proteins
Nitrotyrosine (free or in proteins)	A	Damaged proteins
Protein carbonyls/OxyBlot^R^ technique	A	Largely HNE and related alpha, beta-unsaturated aldehydes conjugated to protein
Dityrosine	A	Damaged proteins
Superoxide	B	Oxidized Ethidium - requires High Performance Liquid Chromatography HPLC
Glutathione/GSH/GSSG	B	Transient changes - requires time course
Lipid peroxides/lipid peroxidation/lipid hydroperoxides	B	Transient changes - requires time course
Peroxynitrite	B	Transient changes - requires time course
Hydrogen peroxide	B	Transient changes - requires time course
Acrolein	X	Metabolism can also produce
Ascorbic acid/vitamin C	X	Diet alters in humans
Cysteine	X	Diet alters
Malondialdehyde/MDA/Thiobarbituric acid reacting substances/TBARS	X	Too many non-oxidative stress related reactions produce TBARS, including metabolism
Nfe2l2/Nrf2/Heme Oxygenase/Peroxiredoxin/Thioredoxin/Thoredoxin Reductase 1/NQO1/GCLC/HMOX1/HO-1/PRDX1/TXN1/TXNRD1	X	Not necessarily caused by oxidative stress
Antioxidant response element/electrophilic response element/ARE/EpRE --> Binding/activation/induction	X	Not necessarily caused by oxidative stress
Tocopherol/vitamin E	X	Diet alters
Total antioxidant capacity/Total antioxidant status	X	Meaningless in biological context
DCFDH	X	Measures iron dependent oxidation of dye
Other fluorescent dyes	X	Lack of specificity for real time measurement

**Rating categories of oxidative stress biomarkers:**

A = useful endpoint for *in vitro* and *in vivo.*

B = useful for *in vitro* with a real time course only.

X = not a valid measure of oxidative stress.

Increases in category “A” biomarkers indicate oxidative damage as validated by previous studies, including the biomarker of oxidative stress studies (BOSS) (Jeong et al., 2018, Kadiiska et al., 2005a, Kadiiska et al., 2005b, Kadiiska et al., 2011, Kadiiska et al., 2013, Kadiiska et al., 2015 Apr). Biomarkers in Category “B” are indicators of altered redox homeostasis and are insufficient as markers of oxidative damage. Studies using methods that are not specific to category “A” or “B” biomarkers, such as the immunoassay for 15F2t-IsoProstane or ethidium fluorescence without HPLC, are considered to have a probably high risk of bias.

Table 1 lists all biomarkers with their respective classifications.

Fig. 1 presents criteria related to oxidative stress biomarkers will be used for the selection of studies.

**Exclusion criteria for the full text-screening will be prioritized as follows:**

1. **Not eligible population.**
2. **Have applied exposure signals with more than 10% of the total signal energy outside the considered frequency range 100 kHz–300 GHz.**
3. **Have applied exposure with a small exposure contrast which we do not consider relevant.**
4. **No separate reference group that is sham-exposed or not exposed is reported.**
5. **No measurement of oxidative stress is reported.**

**No valid biomarker for oxidative stress is reported.**

Table 2 defines key terms related to oxidative stress.

**Table 2:** Definition of oxidative stress terms.

Term	Definition
**Reactive oxygen species (ROS)**	Reactive oxygen species are a group of molecules derived from oxygen. While they can act as oxidants, they have marked differences in reactivity. These species are often referred to as free radicals, although only some are. The abbreviation ROS is often used erroneously as if it were a molecular entity (Forman et al., 2015).
**Reactive nitrogen species**	Reactive nitrogen species describes molecules derived from nitric oxide, which is a physiologically important compound (Forman et al., 2015).
**Oxidative stress (OS)**	An imbalance between the generation of oxidizing molecules and the ability to eliminate the oxidants or to prevent or repair the resulting damage. The term was first used by Sies in his book, *Oxidative Stress* (Sies, 1985).
**Oxidative damage**	The functional destruction of macromolecules through oxidation. It was first used in examining the injury of erythrocytes caused by autohemolysis and ascorbate (Rotruck et al., 1972).
**Hormesis**	A term used by toxicologists to refer to a biphasic dose response to an environmental agent characterized by a low dose stimulation or beneficial effect and a high dose inhibitory or “toxic effect”(Mattson, 2008).
**Adaptive homeostasis**	“The transient expansion or contraction of the homeostatic range in response to exposure to sub-toxic, non-damaging, signaling molecules or events, or the removal or cessation of such molecules or events” (Davies, 2016).
**Parahormesis**	Adaptive homeostasis that results from the use of nonessential compounds, including polyphenols, which by primarily activating Nrf2, mimic the effect of endogenously produced electrophiles (Ursini et al., 2016).
**Eustress**	Adaptive homeostasis produced by low concentrations of hydrogen peroxide (Sies, 2017).
**Redox state**	A term that has historically been used to describe the ratio of the interconvertible oxidized and reduced form of a specific redox couple. It was originally defined by [free NAD^+^]/[free NADH], but as glutathione became clearly the dominate small molecule involved with reducing hydroperoxides, [GSSG]/2 [GSH] or the redox potential derived from this, became the preferred calculation, where GSH is glutathione and GSSG is glutathione disulfide (often incorrectly called oxidized glutathione). However, “at best, the [glutathione] redox potential might be useful as an analytical tool to disclose disturbances in redox metabolism” because it does not account for synthesis or other uses of GSH (Flohé, 2013).

#### 3.1.5 Types of studies

Only experimental studies (i.e. studies with primary data reported in the publication), *in vivo* or *in vitro,* exploring associations between controlled exposures to RF-EMF and effects on predefined markers of oxidative stress and damage will be included. These will be studies performed in a controlled laboratory environment where the objects of study, such as animals or cells, are preferably randomly assigned to the exposure and the control condition. We will also include studies that use non-random methods such as alternation or the choice of the researchers.

We will include studies (especially in the case of animal studies) that provide adequate “wash-out” periods after exposure to RF-EMF. Experimental models with wash-out periods have been used previously in studies on the effects of RF-EMF on cognitive function (Regel and Achermann, 2011).

#### 3.1.6 Exclusion criteria

Studies will be excluded if they are

(a) Review articles, opinion papers, case reports, proceedings or abstracts to meetings (i.e. publications without the original data). However, we will review the reference lists of review articles for relevant studies.
(b) Epidemiological studies.
(c) Studies not meeting exposure criteria:
  1. Studies that have applied exposure signals with>10% of the total signal energy outside the considered frequency range 100 kHz − 300 GHz (e.g. pulsed fields, non-sinusoidal fields with dominant frequencies < 100 kHz).
  2. Studies that have applied exposures with a mobile phone not in GSM mode, not on active call, or not controlled by hardware or software. Under these conditions one would expect an extremely small exposure contrast, which we do not consider relevant (see footnote in Section 3.1.2).
  3. Studies that do not report exposure metrics.
  4. Studies without an unexposed or sham-exposed control.
(d) Studies not reporting oxidative stress outcomes.
(e) Studies with data obtained from less than three biological replicates.
(f) Studies evaluating the simultaneous effects of antioxidants and RF-EMF.
(g) Studies where special devices (like radioablation studies) or nanoparticles are injected into humans, animals, organs or cells to enhance RF-EMF effects and trigger oxidative stress.

#### 3.1.7 Types of effect measures

For qualitative synthesis, the systematic review will include any reported quantitative results comparing exposure vs. control or vs. temperature control group. Quantitative is defined as difference in means, percentage-change, or n-fold increase.

*Unit of analysis issues*

*In vitro* studies often have unit of analysis issues. When comparing an exposed Petri dish to an unexposed Petri dish with a specific number of cells or a particular level of confluency each, the cells in each Petri dish will not be independent but the outcomes in these cells will be related to a certain extent. Statistical theory requires the units of analysis to be independent. If the units are clustered, the analysis must be adjusted for the clustering between the units of analysis. If the authors have not adjusted for clustering, we will adjust the effect sizes as recommended in the Cochrane Handbook (Higgins et al., 2019).

### 3.2 Information sources and search strategy

The search strategy combines controlled vocabulary and keyword terms related to the exposures and outcomes in the PECO(S) statement above. The terms were adjusted to achieve a balance of precision and recall in results, and a pre-determined set of on-target articles was used to measure search strategy quality. The following databases will be searched: PubMed, Embase, Web of Science Core Collection, Scopus, and the EMF-Portal. The reference lists of included studies and retrieved review articles will also be manually searched. All search results will be imported into the reference management software EndNote, which will be used for removing duplicate articles. The search strings for each database are documented in Appendix 1.

Results from our searches will be supplemented by documents retrieved as part of an on-going WHO RF-EMF scoping review.

### 3.3 Study selection

After duplicate removal, the search results will be imported into DistillerSR for study selection. Title and abstract screening will be conducted independently by two reviewers and supplemented by DistillerSR's Artificial Intelligence (AI) software.

Studies remaining after title and abstract screening will be screened by two independent reviewers based upon their full texts. Studies excluded at the full-text screening step will be included in an appendix to the published systematic review, along with the reasons for their exclusion.

If the results from a study are duplicated in more than one article, these articles will be treated as a single study. During both screening steps, disagreements between the two reviewers will be resolved by discussion. If no consensus can be reached, a third reviewer will be consulted.

The selection process will be documented in a study flow diagram according to the PRISMA reporting guidelines (Liberati et al., 2009).

### 3.4 Data extraction

For each study included in the current review, a standard set of data (see Table 3) will be extracted, including the supplemental information provided with each publication. We will try to obtain numerical data whenever possible, and will use the online tool “WebPlot Digitizer” https://automeris.io/WebPlotDigitizer/ to retrieve numerical values from charts and graphs. Using data extraction forms in DistillerSR, one reviewer will extract and record the relevant features of each eligible study. A second reviewer will check the extracted study information against the accompanying article(s) for completeness and accuracy as a quality control measure. If one of the authors of the review is also an author of an included study, we will make sure that this author will not extract data from their own study.

**Table 3:** Overview of extracted data for *in vivo* and *in vitro* studies.

Extracted information	Description/Items
**Citation**	Include details such as journal, title, author, volume, page numbers, etc.
**Year**	Year of publication
**Objective**	Describe the study objective as stated by the authors

**Study details:**	
Sponsorship source	Industry, government, independent (NGO), mixed (firewall), unknown
Declaration of Competing Interest	Yes/no
Country	
Year of publication	Print/latest
Setting	Laboratory or another place
Comments	Multicenter study

**Author contact details:**	
Authorś names	Initials
Corresponding author	
Institutions	All; including author affiliations
E-Mail	Corresponding author
Address	Corresponding author

**Population:**	
> Population type	Human/animal species
> Details	Describe details of animal strain, cell culture, etc. including age, sample preparation, …
> Status of organism	*In vivo*/*in vitro*
> Sample type	Cell/tissue/blood sample/organoid
> Sample size exposure group	Number of samples in exposure group
> Sample size control group	Number of samples in control group
**Control group** (=Comparator)	Sham-exposed/non-exposed
**Control group setup**	Was control group independent of exposure group or was exposure used as own control group (e.g. unexposed at time 0)?
**Random assignment**	Were samples randomly assigned to groups? Yes/No

**Exposure:**	
> Type of Exposure	Uniform (e.g. whole body)/non-uniform (e.g. partial body) + free field for description on non-uniformity (e.g. exposed body part, homogenous exposure only in the center of the petri dish, etc.)
> Exposure system	Details of exposure system
> EM field parameters	Details of field parameters
> Frequency	Frequency and/or frequency range of applied EMF
> Continuous wave (CW)/intermittent	Exposure (within one session) applied continuously or intermittently (with ON/OFF intervals to be specified)
> Modulation	Modulation type (amplitude modulation (AM)/frequency modulation (FM)/pulsed, etc.) and related details (e.g. pulse duration and repetition rate)
> Exposure protocol	Numeric value in hours (for *in vitro* studies) or hours per day and days per week and number of weeks (or lifelong) for *in vivo* studies
Details of external exposure metric	Reported or not + details, in particular if reported values are time-, spatial- surface average, or respective peak values
Incident power density	Numeric value in W/m^2^ or n.a.
External electric field strength	Numeric value in V/m or n.a.
External magnetic field strength	Numeric value in A/m or n.a.
External magnetic flux density	Numeric value in µT or n.a.
> Details of tissue internal exposure metric	Reported or not + details, in particular if reported values are time-/spatial-/surface average, or respective peak values
Local specific absorption rate	Numeric value in W/kg or n.a.
Whole body specific absorption rate	Numeric value in W/kg or n.a.
Transmitted power density	Numeric value in W/m^2^ or n.a.
Internal electric field strength	Numeric value in V/m or n.a.
Internal magnetic field strength	Numeric value in A/m or n.a.
Internal magnetic flux density	Numeric value in µT or n.a.
Exposure validation	Description of exposure assessment details
Continuous monitoring of exposure	Yes/No
Age/weight at exposure	Yes/No
Uncertainty assessment	Yes/No + free field for details
Expanded (k = 2) uncertainty of tissue internal metric	Numeric value in %
Temperature assessment	Reported or not + details
Exposure-induced temperature elevation(s)	Numeric value(s) in K + expanded (k = 2) uncertainty + free field for additional details (location of measurement(s))
Temperature control group (*in vitro* only)	Reported or not + details

**Biomarker:**	Name of biomarker
> Value	Value of biomarker measurement (e.g. mg/l, %.)
> Measurement	Describe details of measurement of biomarker: which method was used)
> Standards (in case there is one)	Standard curve, quantitative measurement, correlation coefficient (for standards)
> Blinding	Yes/No
> Experimental set-up	Yes/No
> Type of sample	Tissue, body fluid (e.g. blood, urine, serum, plasma), cell type

**Results:**	
> Results reported	Chart or graphs/pictures/table/text/raw data
> Measure of result	Mean/median/percentage change compared to control/percentage cells/…
> Measure of Variation of Result	Standard deviation/standard error/interquartile range/individual data reported
> Study-level^5^: Result	Numeric or not quantified
> Study-level: Variation of result	Numeric or not quantified
> Exposure group: Result	Numeric or not quantified
> Exposure group: Variation of result	Numeric or not quantified
> Control group: Result	Numeric or not quantified
> Control group: Variation of result	Numeric or not quantified
> Temperature Control group: Result (if applicable)	Numeric or not quantified
> Temperature Control group: Variation of result (if applicable)	Numeric or not quantified

Study-level refers to reported results summarizing the observed effect between two (or more) groups.

*Dealing with missing data*

If data necessary for our analysis are missing from an article, we will attempt to calculate these from other data reported in the same article.

For articles published in the last ten years, we will contact authors to request missing data, sending up to two reminders and waiting fourteen days for a response. The communication with authors will be reported in supplements to the data extraction table. When relative values are given, the absolute value of the control should be given whenever possible.

### 3.5 Risk of bias assessment

The risk-of-bias assessment will be based on the “Risk of Bias (RoB) Rating Tool for Human and Animal Studies” developed by the NTP Office of Health Assessment and Translation (OHAT) (NTP-OHAT, , 2019, Rooney et al., 2014 Jul, Rooney et al., 2016). Studies will be assessed individually across six domains, with detailed criteria elaborated for each domain in the form of risk-of-bias questions specific for each type of study design. In case a study uses a crossover design (e.g. in human experimental studies) we will ask whether an equal proportion of participants is allocated to any study group to prevent a period effect (Higgins, 2016). Additionally, we will assess whether there is sufficient time for carry-over effects to disappear before outcomes are assessed in the second period (Higgins, 2016).

As we are dealing with *in vivo* and *in vitro* data, our OHAT risk-of-bias tool has separate sections for each of these types of studies. The risk-of-bias questions will cover the following domains: selection bias, performance bias, attrition/exclusion bias, detection bias, selective reporting bias, and any potential additional bias impacting internal validity, which we describe below in detail using the questions from the OHAT handbook. We will adapt the tier-criteria system from the OHAT handbook (NTP-OHAT, 2019)), using the confounding, exposure, and outcome domains as key criteria for the risk of bias rating. This means that we will use three tier categories. Studies will get a “definitely low” or “probably low” tier 1 risk of bias if they score a low risk of bias rating in all three key domains and most of the other domains. We will use tier 3 “definitely high” or “probably high” risk of bias for studies that score a high risk of bias rating in all three key domains and most of the other domains. Studies that do not receive a tier 1 or tier 3 rating will be rated as tier 2. We will follow the recommendations from the OHAT handbook and consider the following domains and questions:

**Selection bias**

1. Was the administered dose or exposure level adequately randomized?

This question deals with the randomization of the experimental set-up and will be applied as described in the original OHAT tool (NTP/OHAT, 2015).

2. Was allocation to study groups adequately concealed?

This question deals with the concealment of the experimental conditions. The research personnel should not know to which experimental groups humans, animals, or cells were allocated. This domain will be applied as described in the OHAT handbook.

3. Did selection of study participants result in the appropriate comparison groups? (Not applicable for our review)
4. Did study design or analysis account for important confounding and modifying variables? (Not applicable for our review)

Questions 3 and 4 of the OHAT risk of bias tool do not apply in the study designs we are evaluating.

**Performance bias**

5. Were experimental conditions identical across study groups?

This question has been slightly adapted to focus on whether sham-exposed subjects (humans, animals, or cells) were exposed to the same conditions as exposed subjects, apart from the actual exposure. For example, were sham-exposed subjects exposed to the same temperature, atmosphere, and vibrations as test subjects during RF-EMF exposure?

6. Were the research personnel blinded to the study group during the study?

In the case of *in vivo* studies with humans, this question is about whether the research personnel and the human subjects know whether which subjects are exposed to RF-EMF. For *in vivo* studies with animals and *in vitro* studies with cells, we want to determine if the research personnel knew whether animals or cells were exposed to RF-EMF.

**Attrition/Exclusion bias**

7. Were outcome data complete without attrition or exclusion from analysis?

We added minor adaptations to this question to reflect specific aspects of the study types.

**Detection bias**

8. Can we be confident in the exposure characterization?

With this question, we will assess the confidence in the reported exposure levels inside samples and tissues. Furthermore, we want to determine if the exposure contrast is sufficient for RF-EMF-induced effects. This question has been modified to address possible issues that can lead to a wrong exposure characterization.

9. Can we be confident in the outcome assessment?

This question has been modified to address the specificity and sensitivity of biomarkers of oxidative stress, the use of standards in biochemical assays, information enabling the reproduction of experimental conditions, and the characterization of study population.

**Selective reporting bias**

10. Were all measured outcomes reported?

This question will be applied according to the original OHAT handbook guidelines.

**Potential other bias**

11. Are there additional threats to internal validity?

As temperature might affect markers of oxidative stress, we will address whether exposure-related temperature changes have been adequately assessed.

Each of the six domains mentioned above is rated as “definitely low risk of bias”, “probably low risk of bias”, “probably high risk of bias”, “definitely high risk of bias”, or no information available. For the overall assessment of the risk of bias, we will use a 3 tier system follwing the OHAT recommendations (NTP-OHAT, 2019). Studies will get a “definitely low” or “probably low” tier 1 risk of bias, if they score a low risk of bias rating in all three key domains and most of the other domains. We will use tier 3 “definitely high” or “probably high” risk of bias for studies that score a high risk of bias rating in all three key domains and most of the other domains. Studies that do not receive a tier 1 or tier 3 rating will be rated as tier 2.

Risk of bias domains will be independently assessed by two reviewers following the criteria outlined in Appendix B, with disagreements handled by the review team by consensus.

If one of the authors of the review is also an author of an included study, we will make sure that this author does not assess the risk of bias of their own research.

### 3.6 Summary measures and synthesis of results

#### 3.6.1 Quantitative synthesis (Meta-analysis)

If studies are considered sufficiently similar (e.g. dealing with the same animal species or cell lines from the same organ), we will conduct a *meta*-analysis. If a *meta*-analysis is not possible, we will perform a narrative synthesis of the results. The criterion “sufficiently similar” presents a particular difficulty in our case, as there are many different biomarkers of oxidative stress and even the same biomarker of oxidative stress can be measured in different ways. In many cases, combining different biomarkers of oxidative stress or different measurement methods, even for the same biomarker, may not be possible.

In cases with a continuous measurement scale of the biomarker and independent groups (exposed versus control group), standardized differences of mean values (Hedges’ g), or standardized mean differences (SMD) will be used as an effect measure. If this effect measure is not directly reported, an attempt will be made to derive the Hedges’ g from other reported results (e.g. mean, median, standard deviation, standard error, interquartile range, sample size).

If the outcome is dichotomous and the results are reported as a percentage or count per group, the corresponding relative risk (RR) will be derived as an effect measure. If this is not possible, the effect measures will either be calculated from the raw data included in the publication or supplemental material or the publication will not be included in the *meta*-analysis.

Considering the diversity of study designs, a DerSimonian-Laird random-effect model (DerSimonian and Laird, 1986) will be applied to calculate the pooled effect size measure and the corresponding confidence interval. For testing the pooled effect size measure from the random effect models, p-values smaller than α = 0.05 will be considered statistically significant. Individual study results as well as the pooled results will be depicted as forest plots. For further association analyses, appropriate visualization techniques such as scatterplots or boxplots will be applied.

If enough data are available, we will calculate the effect per exposure category versus control and perform an exposure–response analysis (Crippa and Orsini, 2016, Orsini et al., 2012 Jan 1). For studies with definitely and probably low risk of exposure bias, we will base the exposure contrast on differences in meaningfully stratified tissue internal exposure metrics.

#### 3.6.2 Qualitative synthesis

If a *meta*-analysis is not possible, we will perform a qualitative synthesis in which the effects of studies will be systematically aggregated in a narrative assessment by translating the results into categories of changed/unchanged or increased/decreased/stable. (Murad et al., 2017).

The number of publications per category will be depicted as tables and figures. Analysis of these results will be done for studies considered sufficiently homogeneous to be combined (see the section on heterogeneity below). For visualization, suitable methods such as scatterplots or bar charts will be used. We will not pool data from different species or biological set-ups. We will not pool human and animal data and we will not pool *in vivo* and *in vitro* data. As the main objective of the WHO reviews is to inform health policy and research, we will prioritize the data from different sources. Data from *in vivo* animal studies are of less relevance than studies with data from humans. We will use *in vitro* data mainly to elucidate possible mechanisms of RF-EMF on oxidative stress.

#### 3.6.3 Heterogeneity

We will only combine studies, narratively or in a *meta*-analysis, that are considered sufficiently similar in all PECO(S) elements. We consider the following PECO(S) elements as different:

- Designs of *in vivo* studies and *in vitro* studies
- Objects of study in *in vivo* studies of different species
- Objects of study in *in vitro* studies such as cells or cell structures as listed under criteria for including studies
- Biomarkers that relate to a different underlying mechanism/biochemical target such as biomarkers of DNA or RNA oxidation (we will not pool RNA and DNA data), biomarkers of lipid peroxidation, or biomarkers related to formation of disulfides. Within these categories, biomarker outcomes will be considered similar enough to be combined.
- Exposure metrics, except if conversion is possible^4^
- Whole body and partial body exposure in animal and human studies
- Type of comparator (control or temperature control)

When a *meta*-analysis is possible, we will examine the amount of statistical variation between study results by the Tau^2^ estimate. The Tau^2^ estimate will also be used to calculate prediction intervals on the populations effect size measure (Higgins et al., 2009). In addition, we will report the heterogeneity measure I^2^ (Higgins and Thompson, 2002, Higgins et al., 2003 Sep 4). In case of I^2^ > 75%, studies will not be combined.

If enough data are available, possible sources of between-study heterogeneity will be investigated by performing subgroup analyses or *meta*-regression considering the below listed aspects:

• Study quality (Risk of Bias rating, following the OHAT handbook (NTP-OHAT, 2019) we will look for the largest contrast)):
  o Low or probably low
  o High or definitely high
• Funding source:
  o Not involving industry
  o Involving industry
  o Mixed funding (firewall prevents influence of industry)
  o Unknown
• Exposure protocol within stratified exposure categories
• Objects of different age (*in vivo* studies, *in vitro* studies only if it is a research question)
  o Young
  o Old

We will use the definition in Table 4 for humans (UN, 1982), mice (Foster et al., 2014) and rats (Andreollo et al., 2012) in our review:

**Table 4:** Definition of age groups for humans, mice and rats.

Species	Infancy	Youth	Young adult	Middle Adult	Old Adult	Elderly
Human (years)	< 1	1–14	15–24	25–44	45–64	> 64
Mouse (months)	< 1	1–2	3–6	7–10	11–17	> 18
Rat (months)	< 1	1–6	7–12	13–18	19–26	> 26

For other mammals we will use the median across all studies for the respective age groups.

• Objects of different sex:
  o Female (for *in vitro* this means cells isolated from female animals and humans)
  o Male (for *in vitro* this means cells isolated from male animals and humans)
• Objects of different health status:
  o Healthy (for *in vitro* this means cells isolated from healthy animals or humans)
  o Diseased (for *in vitro* this means cells isolated from diseased animals or humans)
• Objects of different body weight (only *in vivo* studies)
  o Below median body weight
  o Above median body weight

The median body weight will be calculated within the same species and across all studies. In fact, while higher than average body mass does not necessarily have implications for safety, body mass in animal studies is important from the perspective of dosimetry, i.e. animals with different body masses will absorb RF-energy differently.

• EMF induced heating
  o Heating
  o No heating
• Exposure frequency
  o 100 kHz–10 MHz (below beta dispersion)
  o 1 MHz–100 MHz (feasible range for Radical Pair Mechanism)
  o 100 MH–10 GHz
  o 6 GHz–300 GHz (only superficial absorption)
• Exposure level
  o Non-overlapping clusters of exposure levels (at maximum three clusters)
• Different types of signals:
  o CW-signals
  o Signals with low-frequency characteristics of the signal envelope, as caused by modulation, access, duplex, or power control schemes of wireless communication technologies, e.g., amplitude shift keying (ASK), time division multiple access, time division duplex.
    • Fundamental frequency of the envelope: 0–1 kHz,
    • Fundamental frequency of the envelope: 1 kHz − 1 MHz,
    • Fundamental frequency of the envelope > 1 MHz
  o Time course of exposure
    • Signals with intermittent pattern, i.e., if one of the above signal types is applied according to a specific time pattern, e.g. several seconds/minutes ON/several seconds/minutes OFF
  o Signals without intermittent pattern
  o Any other types of signals

#### 3.6.4 Sensitivity analyses

A sensitivity analysis will be performed to investigate the impact and robustness of the assumptions made in our study protocol.

#### 3.6.5 Publication bias

Depending on the results of the Egger’s linear regression test for funnel plot asymmetry and the number of studies included in the *meta*-analysis, appropriate statistical methods (e.g. Duval and Tweedie, 2000) will be applied to correct pooled results for potentially missing publications.

### 3.7 Assessing evidence quality

We will grade the quality of the evidence following the GRADE (Grading of Recommendations Assessment, Development and Evaluation) standards (Guyatt et al., 2011 Apr 1, Hoffmann et al., 2017, Rooney et al., 2014 Jul). We will assign an overall certainty rating across all studies based on four levels of certainty: very low, low, moderate, and high (Higgins et al., 2019). For rating, we will use the five standard GRADE domains: study limitations (including the bias across all studies), inconsistency of results, indirectness of evidence, imprecision of results, and high probability of publication bias. We will use the online tool GRADEpro https://gradepro.org/ for this. The grading will start by initially assigning a high level of certainty to the evidence in studies and then downgrade the evidence using the five GRADE domains.

The assessment of the evidence in GRADE will be done at the level of endpoints (e.g. biomarkers of oxidative stress).

We will rate the certainty of all conclusions regarding the influence of RF-EMF on biomarkers of oxidative stress using the GRADE approach.

For non-quantified results, we will perform a structured analysis of the possible risk of publication bias. For quantified study results for which an effect measure can be extracted or derived, (see Section 3.1.7), the risk of publication bias will be assessed using funnel plots (if at least 10 studies are available) and the Egger’s linear regression test for asymmetry. The funnel plot will display the effect size measure on the x-axis and the standard error on the y-axis.

The GRADE approach will be performed by two reviewers, each reviewer will perform a GRADE analysis using GRADEpro. In case of disagreements, the GRADE assessment will be discussed with the experts of the review team.

## Funding

This project is funded by the 10.13039/100004423World Health Organization, Geneva, Switzerland under grant 2020/1048580-0.

## CRediT authorship contribution statement

**Bernd Henschenmacher:** Project administration, Funding acquisition, Writing – review & editing. **Annette Bitsch:** Conceptualization, Writing-review & editing. **Tonia de las Heras Gala:** Writing – review & editing. **Henry Jay Forman:** Writing – original draft. **Athanassios Fragoulis:** Writing – original draft. **Pietro Ghezzi:** Writing – original draft. **Rupert Kellner:** Methodology, Writing-review & editing. **Wolfgang Koch:** Writing-original draft. **Jens Kuhne:** Writing – original draft. **Dmitrij Sachno:** Software, Validation. **Gernot Schmid:** Writing – original draft. **Katya Tsaioun:** Methodology. **Jos Verbeek:** Methodology, Writing – original draft. **Robert Wright:** Resources, Software, Writing - review & editing.

## Declaration of Competing Interest

The authors declare that they have no known competing financial interests or personal relationships that could have appeared to influence the work reported in this paper.

Bernd Henschenmacher has been working for the German Federal Office for Radiation Protection in the Division of Effects and Risks of Electric, Magnetic and Electromagnetic Fields from 2019 until 2020 and since 2020 at the Competence Center for Electromagnetic Fields.

Jens Kuhne has been working for the German Federal Office for Radiation Protection in the Division of Effects and Risks of Electric, Magnetic and Electromagnetic Fields from 2017 until 2020 and since 2020 at the Competence Center for Electromagnetic Fields.

Tonia de las Heras Gala has been working for the German Federal Office for Radiation Protection in the Division of Radiation Epidemiology and Risk Assessment since 2019.

Gernot Schmid has been working for Seibersdorf Laboratories (formerly Austrian Research Centers) in the working group Electromagnetic Compatibility since 1997.

## Appendix A Supplementary material

Supplementary data 1 Online appendix A1. Search strategy for PubMed.

Supplementary data 2 Online appendix A2. Search strategy for Embase.

Supplementary data 3 Online appendix A3. Search strategy for Scopus.

Supplementary data 4 Online appendix A4. Search strategy for Web of Science Core Collection.

Supplementary data 5 Online appendix A5. Search strategy for the EMF-Portal.

Supplementary data 6 Appendix B Instructions for assessment of risk of bias in individual studies.

Supplementary data 7 Online appendix C1 GRADE tool for in vitro studies.

Supplementary data 8 Online appendix C2 GRADE tool for animal studies.

## Footnotes

1 We will exclude medical applications from this review see the section 3.1.6 subpoint f.

2 Strictly speaking, exposure–response relationships are evaluated in this SR. Dose (e.g. applied/absorbed amount of compound of per unit mass [toxicology] or applied/absorbed energy per unit mass [ionizing radiation]) is a quantity that typically describes the accumulated exposure of the study object. According to current knowledge, EMF or EMF-induced effects do not accumulate in study objects, which is why exposure–response relationships are of interest. By taking exposure time into account, the conversion of exposure–response relationships into dose–response relationships is possible.

3 Data from the literature (Jalilian et al., 2019) suggest that RFE-MF fields of approximately 0.1–0.3 V/m (corresponding to a PD of 25 – 250 µW/m2) represent a reasonable range for typical indoor RF background exposure in a laboratory environment. From other studies (Christ et al., 2006a, 2006b; Schmid and Kuster, 2015), it can be derived that such background exposure levels may lead to SAR values inside superficial tissue layers and cell cultures on the order of several tens to several hundreds of µW/kg, and that an RF source with a low directivity antenna (e.g. a mobile phone) operated within a few centimetres of the body or biological sample may cause SAR values in superficial layers in the range of several tens to lower hundreds W/kg per watt transmitting power. Therefore, a mobile phone in GSM operating mode with an active call (peak transmitting power in the range approx. of imately 1 mW up to 2W) in close proximity to a body/tissue/sample is expected to cause substantially higher (approx.by an imate factor of 100–1000) temporal peak SAR than typical RF background exposure, even when the transmitting power of the mobile phone (in GSM mode) was not controlled by the experiment, but autonomously controlled by the network. Despite the expected high variation of exposure in such cases, we will therefore also include studies that used a mobile phone in GSM mode with an active call at distances equal to or<3 cm from the body/tissue/samples in a field source, provided that evidence is given that the active call was maintained throughout the experiment and that the control group (sham exposed group) was treated with the mobile phone switched off.UMTS or LTE networks offer a more efficient power adaptation, leading to typically lower exposures compared to GSM mode (Kühn and Kuster, 2012; Joshi et al., 2017). Hence, a sufficient exposure contrast may not exist in studies using non-GSM devices, whose output power is not controlled by the experiment but by the network. Therefore, such studies will not be included.

## Footnotes

4 Studies which do not explicitly provide a quantitative exposure metric or provide metrics that are clearly not related to the experiment (e.g. stating the SAR derived in compliance testing without taking into account the power control of the device during the experiment), are treated as the same class, no metric given.
