# Supplementary material for: The effect of radiofrequency electromagnetic fields (RF-EMF) on biomarkers of oxidative stress in vivo and in vitro: A protocol for a systematic review
Source: Environ Int. 2022 Jan;158:106932. doi: 10.1016/j.envint.2021.106932 (PMC8668870; doi:10.1016/j.envint.2021.106932)
Supplement: Supplementary data 1 — Online appendix A1. Search strategy for PubMed. [file mmc1.pdf]

PubMed Search

Concept 1 – Oxidative Stress

"Oxidative Stress"[Mesh:NoExp] OR "Oxidative Stress*"[tw] OR "Oxidant Stress*"[tw] OR "Protein Carbonylation"[Mesh] OR "Protein Carbonylation*"[tw] OR "Carbonylated Protein Formation"[tw] OR "Protein Carbonyl Formation"[tw] OR "Reactive Oxygen Species"[Mesh:NoExp] OR "Reactive Oxygen Species"[tw] OR "Reactive Oxygen Metabolite*"[tw] OR "Active Oxygen"[tw] OR "Oxygen Radical*"[tw] OR "Pro-Oxidant*"[tw] OR "Hydroxyl Radical"[Mesh] OR "Hydroxyl Radical*"[tw] OR "Hydroxyl Free Radical*"[tw] OR "OH Radical*"[tw] OR "3352-57-6"[rn] OR "Peroxides"[Mesh:NoExp] OR "Organic Peroxide*"[tw] OR Peroxides[tw] OR "14915-07-2"[rn] OR "Hydrogen Peroxide"[Mesh] OR "Hydrogen Peroxide"[tw] OR H2O2[tw] OR "Hydrogen Dioxide"[tw] OR Hydrogenperoxide[tw] OR Hydroperoxide*[tw] OR "7722-84-1"[rn] OR "Lipid Peroxides"[Mesh] OR "Lipid Peroxide*"[tw] OR Lipoperoxide*[tw] OR Lipohydroperoxide*[tw] OR "15 Hydroperoxy 5,8,11,13 Eicosatetraenoate"[tw] OR "15 Hydroperoxy 5,8,11,13 Eicosatetraenoic Acid"[tw] OR "15 Hydroperoxy 5,8,11,13 Icosatetraenoic acid"[tw] OR "15 Hydroperoxyarachidonate"[tw] OR "15 Hydroperoxyarachidonic Acid"[tw] OR "15 Hydroperoxy Arachidonic Acid"[tw] OR "15 Hydroperoxyeicosa 5,8,11,13 Tetraenoic Acid"[tw] OR "15 Hydroperoxyeicosatetraenoic Acid"[tw] OR "15 Hydroperoxy Eicosatetraenoic Acid"[tw] OR "15 Hydroperoxyicosatetraenoic Acid"[tw] OR "67675-14-3"[rn] OR "5 HPETE"[tw] OR "5 Hydroperoxy 5,8,11,14 Eicosatetraenoic Acid"[tw] OR "5 Hydroperoxy 6,8,11,14 Eicosatetraenoate"[tw] OR "5 Hydroperoxy 6,8,11,14 Eicosatetraenoic Acid"[tw] OR "5 Hydroperoxy 6,8,11,14 Icosatetraenoic Acid"[tw] OR "5 Hydroperoxyarachidonic Acid"[tw] OR "5 Hydroperoxyeicosa 5,8,11,14 Tetraenoic Acid"[tw] OR "5 Hydroperoxyeicosa 6,8,11,14 Tetraenoic Acid"[tw] OR "5 Hydroperoxyeicosatetraenoic Acid"[tw] OR "5 Hydroperoxyicosa 6,8,11,14 Tetraenoic Acid"[tw] OR "5 Hydroperoxyicosatetraenoic Acid"[tw] OR "5 Hydroperoxy Icosatetraenoic Acid"[tw] OR "74581-83-2"[rn] OR "Lipid Peroxidation"[Mesh] OR "Lipid Autooxidation*"[tw] OR "Lipid Autoxidation*"[tw] OR "Lipid Peroxidation*"[tw] OR Lipoperoxidation[tw] OR "Superoxides"[Mesh] OR Superoxide*[tw] OR Superoxyde*[tw] OR "11062-77-4"[rn] OR "Peroxynitrous Acid"[Mesh] OR "Peroxynitrous Acid*"[tw] OR Peroxynitrite*[tw] OR Peroxonitrite*[tw] OR "14691-52-2"[rn] OR "8 Hydroxy 2' Deoxyguanosine"[Mesh] OR "8 Hydroxy 2' Deoxyguanosine"[tw] OR 8OHdG[tw] OR "8-Hydroxydeoxyguanosine"[tw] OR "8-Oxo-2'-Deoxyguanosine"[tw] OR "2'-Deoxy-8-Oxoguanosine"[tw] OR "8-oxodG"[tw] OR "8-oxodGuo"[tw] OR "8-oxo-dG"[tw] OR "8-OH-dG"[tw] OR "8-Oxo-Deoxyguanosine"[tw] OR "8-oxo-dGuo"[tw] OR "8-Oxo-7-Hydrodeoxyguanosine"[tw] OR "8-Oxo-7,8-Dihydrodeoxyguanosine"[tw] OR "2'-Deoxy-8-Oxo-7,8-Dihydroguanosine"[tw] OR "2'-Deoxy-7,8-Dihydro-8-Oxoguanosine"[tw] OR "7,8-Dihydro-8-Oxo-2'-Deoxyguanosine"[tw] OR "8-Oxo-7,8-Dihydro-2'-Deoxyguanosine"[tw] OR "8-Oxodeoxyguanosine"[tw] OR "Acrolein"[Mesh] OR Acrolein[tw] OR Acroleine[tw] OR Acraldehyde[tw] OR "Ethylene Aldehyde"[tw] OR "Acrylic Aldehyde"[tw] OR "Allyl Aldehyde"[tw] OR Propenal[tw] OR Acrylaldehyde[tw] OR Acrylylaldehyde[tw] OR Aqualin[tw] OR "107-02-8"[rn] OR "Ascorbic Acid"[Mesh] OR "Ascorbic Acid"[tw] OR "Cevitamic Acid"[tw] OR "Vitamin C"[tw] OR Hybrin[tw] OR "Potassium Ascorbate"[tw] OR "Sodium Ascorbate"[tw] OR "134-03-2"[rn] OR "15421-15-5"[rn] OR "50-81-7"[rn] OR "Dehydroascorbic Acid"[tw] OR Dehydroascorbate[tw] OR "Dehydrovitamin C"[tw] OR "490-83-5"[rn] OR "3-chlorotyrosine" [Supplementary Concept] OR "3-chlorotyrosine"[tw] OR "3-chloro-L-tyrosine"[tw] OR "Glutathione"[Mesh] OR Glutathione[tw] OR Glutathine[tw] OR Glutathiol[tw] OR Glutathion[tw] OR "gamma-L-Glutamyl-L-Cysteinylglycine"[tw] OR "gamma-L-Glu-L-Cys-Gly"[tw] OR "gamma Glutamylcysteinylglycine"[tw] OR "L-Glutamyl-L-Cysteinylglycine"[tw] OR GSH[tw] OR "70-18-8"[rn] OR

"4-hydroxy-2-nonenal" [Supplementary Concept] OR "4-hydroxy-2-nonenal"[tw] OR "4-hydroxynon-2-enal"[tw] OR "4-hydroxynonen-2-al"[tw] OR "4-HNE cpd"[tw] OR "4-hydroxy-2,3-nonenal"[tw] OR "4-hydroxynonenal"[tw] OR "4-hydroxy nonenal"[tw] OR "29343-52-0"[rn] OR "75899-68-2"[rn] OR "Isoprostanes"[Mesh:NoExp] OR "F2-Isoprostanes"[Mesh] OR Isoprostane*[tw] OR "Dinoprost"[Mesh] OR Dinoprost[tw] OR "PG F2 alpha"[tw] OR "PGF 2 alpha"[tw] OR "PGF 2a"[tw] OR PGF2a[tw] OR PGF2[tw] OR "Prostaglandin F2alpha"[tw] OR "Prostaglandin F 2alpha"[tw] OR PGF2alpha[tw] OR "Prostaglandin F2"[tw] OR "Prostaglandin F 2 a"[tw] OR "Prostaglandin F 2 alpha"[tw] OR "Prostaglandin F 2a"[tw] OR "Prostaglandin F2a"[tw] OR "Prostin F 2 alpha"[tw] OR "Prostin F2 alpha"[tw] OR "U 14583"[tw] OR U14583[tw] OR "551-11-1"[rn] OR "Malondialdehyde"[Mesh] OR Malondialdehyde[tw] OR "Malonic Dialdehyde"[tw] OR Propanedial[tw] OR Malonyldialdehyde[tw] OR "Malonyl Dialdehyde"[tw] OR Malonaldehyde[tw] OR Malonylaldehyde[tw] OR "542-78-9"[rn] OR "Thiobarbituric Acid Reactive Substances"[Mesh] OR TBARS[tw] OR "thiobarbituric acid" [Supplementary Concept] OR "Thiobarbituric Acid"[tw] OR "2-Mercaptobarbituric Acid"[tw] OR Thiobarbiturate[tw] OR "504-17-6"[rn] OR "Methionine Sulfoxide Reductases"[Mesh] OR "EC 1.8.4.5"[rn] OR "Methionine Sulfoxide Reductase*"[tw] OR "Peptide-Methionine (S)-S-oxide Reductase"[tw] OR "Selenoprotein R"[tw] OR "SelR Protein"[tw] OR "Peptide-Methionine (R)-S-oxide Reductase"[tw] OR "Methionine-R-sulfoxide Reductase*"[tw] OR "Methionine-S-oxide Reductase*"[tw] OR "3-nitrotyrosine" [Supplementary Concept] OR nitrotyrosine[tw] OR "3-mononitrotyrosine"[tw] OR "3-nitro-L-tyrosine"[tw] OR "3604-79-3"[rn] OR "NF-E2-Related Factor 2"[Mesh] OR "Nrf2 protein"[tw] OR "NF-E2-related factor 2"[tw] OR "Nuclear Factor E2-Related Factor 2"[tw] OR "Nfe2l2 Protein"[tw] OR "Nuclear Factor (Erythroid-Derived 2)-Like 2 Protein"[tw] OR "nuclear factor erythroid 2-related factor 2"[tw] OR "Protein Nrf2"[tw] OR "Transcription factor NF-E2 related nuclear factor 2"[tw] OR "Transcription factor Nrf2"[tw] OR "Heme Oxygenase (Decyclizing)"[Mesh] OR "EC 1.14.99.3"[rn] OR "Heme Oxygenase"[tw] OR "Haem Oxygenase"[tw] OR "Hemeoxygenase-1"[tw] OR "9059-22-7"[rn] OR Hsp32[tw] OR "Hsp 32"[tw] OR "HO-1 protein"[tw] OR "heat shock protein 32"[tw] OR "Hmox1 protein"[tw] OR "protein Hmox1"[tw] OR "Peroxiredoxins"[Mesh] OR "Alkylhydroperoxide Reductase*"[tw] OR "EC 1.11.1.15"[rn] OR "Pag protein"[tw] OR Peroxidoxin*[tw] OR "Thiol-Specific Antioxidant Protein*"[tw] OR Peroxiredoxin*[tw] OR PRDX3[tw] OR "proliferation-associated protein"[tw] OR "207137-51-7"[rn] OR "Thioredoxins"[Mesh] OR Thioredoxin*[tw] OR "Trx1 protein"[tw] OR "Trx protein"[tw] OR "52500-60-4"[rn] OR "Txn protein"[tw] OR "Thioredoxin-Disulfide Reductase"[Mesh] OR "EC 1.8.1.9"[rn] OR "Trxr1 protein"[tw] OR "9074-14-0"[rn] OR "NAD(P)H Dehydrogenase (Quinone)"[Mesh:NoExp] OR "EC 1.6.99.2"[rn] OR "NAD(P)H dehydrogenase (quinone)"[tw] OR "diaphorase 4"[tw] OR "NAD(P)H-menadione oxidoreductase"[tw] OR "NAD(P)H: (quinone acceptor) oxidoreductase"[tw] OR "NAD(P)H quinone oxidoreductase"[tw] OR "Quinone Reductase"[tw] OR "DT Diaphorase"[tw] OR "Menadione Reductase"[tw] OR "Vitamin K Reductase"[tw] OR "9032-20-6"[rn] OR "NADPH Dehydrogenase"[Mesh] OR "EC 1.6.99.1"[rn] OR "NADPH Dehydrogenase"[tw] OR "NADP Diaphorase"[tw] OR "NADPH Diaphorase"[tw] OR "Old Yellow Enzyme"[tw] OR "NADP Dehydrogenase"[tw] OR "NADPH Oxidation"[tw] OR "NADPH: (Acceptor) Oxidoreductase"[tw] OR "Nicotinamide Adenine Dinucleotide Phosphate Dehydrogenase"[tw] OR "Nicotinamide Adenine Dinucleotide Phosphate Diaphorase"[tw] OR "Triphosphopyridine Nucleotide Diaphorase"[tw] OR "9001-68-7"[rn] OR "Glutamate-Cysteine Ligase"[Mesh] OR "EC 6.3.2.2"[rn] OR "Glutamate-Cysteine Ligase"[tw] OR "gamma-Glutamyl-Cysteine Synthetase"[tw] OR "Glutamylcysteine Synthetase"[tw] OR "9023-64-7"[rn] OR "Antioxidants"[Mesh] OR Antioxidant*[tw] OR "Anti-Oxidant*"[tw] OR "Antioxidation Agent*"[tw] OR "Antioxidation Product*"[tw] OR Antioxidative[tw] OR Antoxidant*[tw] OR Scavenger*[tw] OR "Scavenging Agent*"[tw]

OR "Antioxidant Response Elements"[Mesh] OR "Electrophile Response Element*"[tw] OR "EpRE binding"[tw] OR "EpRE activation"[tw] OR "EpRE induction"[tw] OR Oxyblot*[tw] OR "Vitamin E"[Mesh:NoExp] OR "Tocopherols"[Mesh:NoExp] OR Tocoferol*[tw] OR Tocopherol*[tw] OR "1406-66-2"[rn] OR "alpha-Tocopherol"[Mesh] OR "Vitamin E"[tw] OR "1406-18-4"[rn] OR "59-02-9"[rn] OR "Alpha Tocopherolquinone"[tw] OR Eutrophyl[tw] OR "Tocopheryl Quinone"[tw] OR Tocopherylquinone[tw] OR Tocoquinone[tw] OR "7559-04-8"[rn] OR "Tocotrienols"[Mesh] OR Tocotrienol*[tw] OR "1721-51-3"[rn] OR "epc k1"[tw] OR "127061-56-7"[rn] OR "14101-61-2"[rn] OR "Uric Acid"[Mesh] OR "Uric Acid"[tw] OR "2,6,8-Trihydroxypurine"[tw] OR "2,6,8 Trioxypurine"[tw] OR Trioxopurine[tw] OR Urate[tw] OR "69-93-2"[rn] OR "dityrosine" [Supplementary Concept] OR dityrosine[tw] OR bityrosine[tw] OR "dihydroethidium" [Supplementary Concept] OR dihydroethidium[tw] OR "104821-25-2"[rn] OR "Reduced Ethidium Bromide"[tw] OR "38483-26-0"[rn] OR "diacetyldichlorofluorescein" [Supplementary Concept] OR diacetyldichlorofluorescein[tw] OR "2',7'-dichlorofluorescin diacetate"[tw] OR "DCFH-DA"[tw] OR "2',7'-dichlorofluorescein diacetate"[tw] OR DCFDA[tw] OR "2',7'-difluorofluorescein"[tw] OR "2044-85-1"[rn]

Concept 2 – EMF Exposure

"Electromagnetic Radiation"[Mesh:NoExp] OR "Electromagnetic Wave*"[tw] OR "Electromagnetic Energ*"[tw] OR "Electromagnetic Radiation*"[tw] OR "Radio Waves"[Mesh] OR "Radio Wave*"[tw] OR Radiowave*[tw] OR "Hertzian Wave*"[tw] OR "High Frequency Wave*"[tw] OR "Short Wave*"[tw] OR "Microwave Field*"[tw] OR "Microwave Radiat*"[tw] OR "Microwave Expos*"[tw] OR "Microwave Irradiat*"[tw] OR "Microwave Range*"[tw] OR "Micro Wave Field*"[tw] OR "Micro Wave Radiat*"[tw] OR "Micro Wave Expos*"[tw] OR "Micro Wave Irradiat*"[tw] OR "Micro Wave Range*"[tw] OR "MW Field*"[tw] OR "MW Radiat*"[tw] OR "MW Expos*"[tw] OR "MW Irradiat*"[tw] OR "MW Range*"[tw] OR "M W Field*"[tw] OR "M W Radiat*"[tw] OR "M W Expos*"[tw] OR "M W Irradiat*"[tw] OR "M W Range*"[tw] OR "EHF Wave*"[tw] OR "Ultrahigh Frequency Wave*"[tw] OR UHF[tw] OR Radiofrequenc*[tw] OR "Radio Frequenc*"[tw] OR "RF Wave*"[tw] OR "RF Field*"[tw] OR "RF Electric Field*"[tw] OR "RF Magnetic Field*"[tw] OR "RF Radiation*"[tw] OR "RF Expos*"[tw] OR "RF EMF"[tw] OR "Millimeter Wave*"[tw] OR "Electromagnetic Fields"[Mesh] OR "Electromagnetic Environment*"[tw] OR "Electromagnetic Field*"[tw] OR "Electromagnetic Phenomen*"[tw] OR Electromagnetics[tw] OR Electromagnetism[tw] OR "Radar"[Mesh] OR Radar[tw] OR "Cell Phone"[Mesh:NoExp] OR "Cell Phone*"[tw] OR Cellphone*[tw] OR "Cellular Phone*"[tw] OR "Cellular Telephone*"[tw] OR "Mobile Phone*"[tw] OR "Mobile Telephone*"[tw] OR "Cordless Phone*"[tw] OR "Car Phone*"[tw] OR "Smartphone"[Mesh] OR Smartphone*[tw] OR "Smart Phone*"[tw] OR iPhone*[tw] OR "i-Phone*"[tw] OR Android[tw] OR "Cell Phone Use"[Mesh] OR "Wireless Technology"[Mesh] OR "Wireless Technolog*"[tw] OR "Wireless Communication*"[tw] OR "Wi-Fi"[tw] OR Wifi[tw] OR "Specific Absorption Rate*"[tw] OR "W/kg"[tw] OR "Global System for Mobile Communication*"[tw] OR "Digital Cellular System*"[tw] OR "Universal Mobile Telecommunication System*"[tw] OR UMTS[tw] OR "Code Division Multiple Access"[tw] OR CDMA[tw] OR WCDMA[tw] OR WiMAX[tw] OR Bluetooth[tw] OR "Total Access Communication System"[tw] OR "Terrestrial Trunked Radio"[tw] OR "Digital Enhanced Cordless Telecommunication*"[tw]

Concept 1 AND **Concept 2**
