# Supplementary material for: The effect of radiofrequency electromagnetic fields (RF-EMF) on biomarkers of oxidative stress in vivo and in vitro: A protocol for a systematic review
Source: Environ Int. 2022 Jan;158:106932. doi: 10.1016/j.envint.2021.106932 (PMC8668870; doi:10.1016/j.envint.2021.106932)
Supplement: Supplementary data 2 — Online appendix A2. Search strategy for Embase. [file mmc2.pdf]

Embase Search

Concept 1 – Oxidative Stress

'oxidative stress'/exp OR 'oxidative stress*':ti,ab,kw OR 'oxidant stress*':ti,ab,kw OR 'protein carbonylation'/exp OR 'protein carbonylation*':ti,ab,kw OR 'carbonylated protein formation':ti,ab,kw OR 'protein carbonyl formation':ti,ab,kw OR 'reactive oxygen metabolite'/exp OR 'reactive oxygen metabolite*':ti,ab,kw OR 'reactive oxygen species ros'/exp OR 'reactive oxygen species':ti,ab,kw OR 'active oxygen':ti,ab,kw OR 'oxygen radical*':ti,ab,kw OR 'pro-oxidant*':ti,ab,kw OR 'hydroxyl radical'/exp OR 'hydroxyl radical*':ti,ab,kw OR 'hydroxyl free radical*':ti,ab,kw OR 'oh radical*':ti,ab,kw OR '3352-57-6':rn OR 'peroxide'/de OR 'organic peroxide*':ti,ab,kw OR peroxides:ti,ab,kw OR '14915-07-2':rn OR 'hydrogen peroxide'/exp OR 'hydrogen peroxide':ti,ab,kw OR 'H2O2':ti,ab,kw OR 'hydrogen dioxide':ti,ab,kw OR hydrogenperoxide:ti,ab,kw OR hydroperoxide*:ti,ab,kw OR '7722-84-1':rn OR 'lipid peroxide'/de OR 'lipid peroxide*':ti,ab,kw OR lipoperoxide*:ti,ab,kw OR 'lipid hydroperoxide'/exp OR lipohydroperoxide*:ti,ab,kw OR '15 hydroperoxy 5, 8, 11, 13 eicosatetraenoate':ti,ab,kw OR '15 hydroperoxy 5, 8, 11, 13 eicosatetraenoic acid':ti,ab,kw OR '15 hydroperoxy 5, 8, 11, 13 icosatetraenoic acid':ti,ab,kw OR '15 hydroperoxyarachidonate':ti,ab,kw OR '15 hydroperoxyarachidonic acid':ti,ab,kw OR '15 hydroperoxy arachidonic acid':ti,ab,kw OR '15 hydroperoxyeicosa 5, 8, 11, 13 tetraenoic acid':ti,ab,kw OR '15 hydroperoxyeicosatetraenoic acid':ti,ab,kw OR '15 hydroperoxy eicosatetraenoic acid':ti,ab,kw OR '15 hydroperoxyicosatetraenoic acid':ti,ab,kw OR '67675-14-3':rn OR '5 hpete':ti,ab,kw OR '5 hydroperoxy 5, 8, 11, 14 eicosatetraenoic acid':ti,ab,kw OR '5 hydroperoxy 6, 8, 11, 14 eicosatetraenoate':ti,ab,kw OR '5 hydroperoxy 6, 8, 11, 14 eicosatetraenoic acid':ti,ab,kw OR '5 hydroperoxy 6, 8, 11, 14 icosatetraenoic acid':ti,ab,kw OR '5 hydroperoxyarachidonic acid':ti,ab,kw OR '5 hydroperoxyeicosa 5, 8, 11, 14 tetraenoic acid':ti,ab,kw OR '5 hydroperoxyeicosa 6, 8, 11, 14 tetraenoic acid':ti,ab,kw OR '5 hydroperoxyeicosatetraenoic acid':ti,ab,kw OR '5 hydroperoxyicosa 6, 8, 11, 14 tetraenoic acid':ti,ab,kw OR '5 hydroperoxyicosatetraenoic acid':ti,ab,kw OR '5 hydroperoxy icosatetraenoic acid':ti,ab,kw OR '74581-83-2':rn OR 'lipid peroxidation'/exp OR 'lipid autooxidation*':ti,ab,kw OR 'lipid autoxidation*':ti,ab,kw OR 'lipid peroxidation*':ti,ab,kw OR lipoperoxidation:ti,ab,kw OR 'superoxide'/exp OR superoxide*:ti,ab,kw OR superoxyde*:ti,ab,kw OR '11062-77-4':rn OR 'peroxynitrous acid'/exp OR 'peroxynitrous acid*':ti,ab,kw OR peroxynitrite*:ti,ab,kw OR peroxonitrite*:ti,ab,kw OR '14691-52-2':rn OR '8 hydroxydeoxyguanosine'/exp OR '2 deoxy 7, 8 dihydro 8 oxoguanosine':ti,ab,kw OR '2 deoxy 8 oxo 7, 8 dihydroguanosine':ti,ab,kw OR '2 deoxy 8 oxoguanosine':ti,ab,kw OR '7, 8 dihydro 8 oxo 2 deoxyguanosine':ti,ab,kw OR '8 hydroxy 2 deoxyguanosine':ti,ab,kw OR '8 hydroxydeoxyguanosine':ti,ab,kw OR 8OHdG:ti,ab,kw OR '8 OH dG':ti,ab,kw OR '8 oxo 2 deoxyguanosine':ti,ab,kw OR '8 oxo 7 hydrodeoxyguanosine':ti,ab,kw OR '8 oxo 7, 8 dihydro 2 deoxyguanosine':ti,ab,kw OR '8 oxo 7, 8 dihydrodeoxyguanosine':ti,ab,kw OR '8 oxodeoxyguanosine':ti,ab,kw OR '8 oxodG':ti,ab,kw OR '8 oxodGuo':ti,ab,kw OR '8 oxo deoxyguanosine':ti,ab,kw OR '8 oxo dG':ti,ab,kw OR '8 oxo dGuo':ti,ab,kw OR 'acrolein'/exp OR acraldehyde:ti,ab,kw OR acrolein:ti,ab,kw OR acroleine:ti,ab,kw OR acrylaldehyde:ti,ab,kw OR 'acrylic aldehyde':ti,ab,kw OR acrylylaldehyde:ti,ab,kw OR 'allyl aldehyde':ti,ab,kw OR aqualin:ti,ab,kw OR 'ethylene aldehyde':ti,ab,kw OR propenal:ti,ab,kw OR '107-02-8':rn OR 'ascorbic acid'/de OR 'ascorbic acid':ti,ab,kw OR 'cevitamic acid':ti,ab,kw OR hybrin:ti,ab,kw OR 'potassium ascorbate':ti,ab,kw OR 'sodium ascorbate':ti,ab,kw OR 'vitamin c':ti,ab,kw OR '134-03-2':rn OR '15421-15-5':rn OR '50-81-7':rn OR 'dehydroascorbic acid'/exp OR 'dehydroascorbic acid':ti,ab,kw OR dehydroascorbate:ti,ab,kw OR 'dehydrovitamin c':ti,ab,kw OR '490-83-5':rn OR '3 chlorotyrosine'/exp OR '3 chlorotyrosine':ti,ab,kw OR

'3 chloro l tyrosine':ti,ab,kw OR 'glutathione'/exp OR 'gamma glutamylcysteinylglycine':ti,ab,kw OR 'gamma-l-glu-l-cys-gly':ti,ab,kw OR 'gamma-l-glutamyl-l-cysteinylglycine':ti,ab,kw OR glutathine:ti,ab,kw OR glutathiol:ti,ab,kw OR glutathion:ti,ab,kw OR glutathione:ti,ab,kw OR GSH:ti,ab,kw OR 'l glutamyl l cysteinylglycine':ti,ab,kw OR '70-18-8':rn OR '4 hydroxynonenal'/exp OR '4 hne cpd':ti,ab,kw OR '4 hydroxy 2 nonenal':ti,ab,kw OR '4 hydroxy 2, 3 nonenal':ti,ab,kw OR '4 hydroxynon 2 enal':ti,ab,kw OR '4 hydroxynonen 2 al':ti,ab,kw OR '4 hydroxynonenal':ti,ab,kw OR '4 hydroxy nonenal':ti,ab,kw OR '29343-52-0':rn OR '75899-68-2':rn OR 'isoprostane derivative'/exp OR isoprostane*:ti,ab,kw OR 'isoprostane f2'/exp OR 'prostaglandin F2 alpha'/exp OR dinoprost:ti,ab,kw OR 'pg f2 alpha':ti,ab,kw OR 'pgf 2 alpha':ti,ab,kw OR 'pgf 2a':ti,ab,kw OR pgf2:ti,ab,kw OR pgf2a:ti,ab,kw OR pgf2alpha:ti,ab,kw OR 'prostaglandin f 2 a':ti,ab,kw OR 'prostaglandin f 2 alpha':ti,ab,kw OR 'prostaglandin f 2a':ti,ab,kw OR 'prostaglandin f 2alpha':ti,ab,kw OR 'prostaglandin f2':ti,ab,kw OR 'prostaglandin f2a':ti,ab,kw OR 'prostaglandin f2alpha':ti,ab,kw OR 'prostin f 2 alpha':ti,ab,kw OR 'prostin f2 alpha':ti,ab,kw OR 'u 14583':ti,ab,kw OR u14583:ti,ab,kw OR '551-11-1':rn OR 'malonaldehyde'/exp OR malonaldehyde:ti,ab,kw OR malondialdehyde:ti,ab,kw OR 'malonic dialdehyde':ti,ab,kw OR 'malonyl dialdehyde':ti,ab,kw OR malonylaldehyde:ti,ab,kw OR malonyldialdehyde:ti,ab,kw OR propanedial:ti,ab,kw OR '542-78-9':rn OR 'thiobarbituric acid reactive substance'/exp OR TBARS:ti,ab,kw OR 'thiobarbituric acid'/exp OR '2 mercaptobarbituric acid':ti,ab,kw OR 'thiobarbituric acid':ti,ab,kw OR thiobarbiturate:ti,ab,kw OR '504-17-6':rn OR 'methionine sulfoxide reductase'/exp OR 'e.c. 1.8.4.5':ti,ab,kw OR 'methionine r sulfoxide reductase*':ti,ab,kw OR 'methionine s oxide reductase*':ti,ab,kw OR 'methionine sulfoxide reductase*':ti,ab,kw OR 'peptide methionine (r) s oxide reductase*':ti,ab,kw OR 'peptide methionine (s) s oxide reductase*':ti,ab,kw OR 'selenoprotein r':ti,ab,kw OR 'selr protein':ti,ab,kw OR '3 nitrotyrosine'/exp OR '3 mononitrotyrosine':ti,ab,kw OR '3 nitro l tyrosine':ti,ab,kw OR nitrotyrosine:ti,ab,kw OR '3604-79-3':rn OR 'transcription factor Nrf2'/exp OR 'NF E2 related factor 2':ti,ab,kw OR 'Nfe2l2 protein':ti,ab,kw OR 'Nrf2 protein':ti,ab,kw OR 'nuclear factor E2 related factor 2':ti,ab,kw OR 'nuclear factor erythroid 2 related factor 2':ti,ab,kw OR 'nuclear factor (erythroid derived 2) like 2 protein':ti,ab,kw OR 'protein Nrf2':ti,ab,kw OR 'transcription factor NF E2 related nuclear factor 2':ti,ab,kw OR 'transcription factor Nrf2':ti,ab,kw OR 'heme oxygenase'/exp OR 'e.c. 1.14.99.3':ti,ab,kw OR 'heme oxygenase':ti,ab,kw OR 'haem oxygenase':ti,ab,kw OR '9059-22-7':rn OR 'heme oxygenase 1'/exp OR 'heat shock protein 32':ti,ab,kw OR 'hemeoxygenase 1':ti,ab,kw OR 'HMOX1 protein':ti,ab,kw OR 'HSP 32':ti,ab,kw OR 'HSP32':ti,ab,kw OR 'protein HMOX1':ti,ab,kw OR 'ho 1 protein'/exp OR 'ho 1 protein':ti,ab,kw OR 'peroxiredoxin'/exp OR 'alkylhydroperoxide reductase*':ti,ab,kw OR 'e.c. 1.11.1.15':ti,ab,kw OR 'Pag protein':ti,ab,kw OR peroxidoxin*:ti,ab,kw OR peroxiredoxin*:ti,ab,kw OR PRDX3:ti,ab,kw OR 'proliferation associated protein':ti,ab,kw OR 'thiol specific antioxidant protein*':ti,ab,kw OR '207137-51-7':rn OR 'thioredoxin'/exp OR thioredoxin*:ti,ab,kw OR 'Trx1 protein':ti,ab,kw OR 'Trx protein':ti,ab,kw OR '52500-60-4':rn OR 'txn protein human'/exp OR 'txn protein':ti,ab,kw OR 'thioredoxin reductase'/exp OR 'e.c. 1.8.1.9':ti,ab,kw OR 'Trxr1 protein':ti,ab,kw OR '9074-14-0':rn OR 'reduced nicotinamide adenine dinucleotide (phosphate) dehydrogenase (quinone)'/exp OR 'diaphorase 4':ti,ab,kw OR 'DT diaphorase':ti,ab,kw OR 'e.c. 1.6.99.2':ti,ab,kw OR 'menadione reductase':ti,ab,kw OR 'NAD(P)H dehydrogenase (quinone)':ti,ab,kw OR 'NAD(P)H menadione oxidoreductase':ti,ab,kw OR 'NAD(P)H: (quinone acceptor) oxidoreductase':ti,ab,kw OR 'NAD(P)H quinone oxidoreductase':ti,ab,kw OR 'quinone reductase*':ti,ab,kw OR 'vitamin k reductase':ti,ab,kw OR '9032-20-6':rn OR 'nad p h dehydrogenase quinone 1'/exp OR 'nadph dehydrogenase quinone 1'/exp OR 'reduced nicotinamide adenine dinucleotide phosphate dehydrogenase'/exp OR 'e.c. 1.6.99.1':ti,ab,kw OR 'NADP

dehydrogenase':ti,ab,kw OR 'NADP diaphorase':ti,ab,kw OR 'NADPH dehydrogenase':ti,ab,kw OR 'NADPH diaphorase':ti,ab,kw OR 'NADPH oxidation':ti,ab,kw OR 'NADPH: (acceptor) oxidoreductase':ti,ab,kw OR 'nicotinamide adenine dinucleotide phosphate dehydrogenase':ti,ab,kw OR 'nicotinamide adenine dinucleotide phosphate diaphorase':ti,ab,kw OR 'old yellow enzyme':ti,ab,kw OR 'reduced nicotinamide adenine dinucleotide phosphate diaphorase':ti,ab,kw OR 'triphosphopyridine nucleotide diaphorase':ti,ab,kw OR '9001-68-7':rn OR 'glutamate cysteine ligase'/exp OR 'e.c. 6.3.2.2':ti,ab,kw OR 'gamma glutamyl cysteine synthetase':ti,ab,kw OR 'glutamate cysteine ligase':ti,ab,kw OR 'glutamylcysteine synthetase':ti,ab,kw OR '9023-64-7':rn OR 'antioxidant'/de OR 'anti oxidant*':ti,ab,kw OR antioxidant*:ti,ab,kw OR 'antioxidation agent*':ti,ab,kw OR 'antioxidation product*':ti,ab,kw OR antioxidative:ti,ab,kw OR antoxidant*:ti,ab,kw OR 'scavenger'/exp OR scavenger*:ti,ab,kw OR 'scavenging agent*':ti,ab,kw OR 'antioxidant responsive element'/exp OR 'electrophile response element'/exp OR 'electrophile response element*':ti,ab,kw OR 'EpRE binding':ti,ab,kw OR 'EpRE activation':ti,ab,kw OR 'EpRE induction':ti,ab,kw OR oxyblot*:ti,ab,kw OR 'tocopherol'/de OR tocoferol*:ti,ab,kw OR tocopherol*:ti,ab,kw OR '1406-66-2':rn OR 'alpha tocopherol'/exp OR 'vitamin E':ti,ab,kw OR '1406-18-4':rn OR '59-02-9':rn OR 'alpha tocopherylquinone'/exp OR 'alpha tocopherolquinone':ti,ab,kw OR eutrophyl:ti,ab,kw OR 'tocopheryl quinone':ti,ab,kw OR tocopherylquinone:ti,ab,kw OR tocoquinone:ti,ab,kw OR '7559-04-8':rn OR 'alpha tocotrienol'/exp OR tocotrienol*:ti,ab,kw OR '1721-51-3':rn OR 'ascorbic acid 2 [3,4 dihydro 2,5,7,8 tetramethyl 2 (4,8,12 trimethyltridecyl) 2h 1 benzopyran 6 yl hydrogen phosphate] potassium'/exp OR 'epc k1':ti,ab,kw OR '127061-56-7':rn OR 'gamma tocotrienol'/exp OR '14101-61-2':rn OR 'total antioxidant capacity'/exp OR 'uric acid'/exp OR '2, 6, 8 trihydroxypurine':ti,ab,kw OR '2, 6, 8 trioxypurine':ti,ab,kw OR 'trioxopurine':ti,ab,kw OR urate:ti,ab,kw OR 'uric acid':ti,ab,kw OR '69-93-2':rn OR 'dityrosine'/exp OR dityrosine:ti,ab,kw OR bityrosine:ti,ab,kw OR 'hydroethidine'/exp OR dihydroethidium:ti,ab,kw OR '104821-25-2':rn OR 'reduced ethidium bromide':ti,ab,kw OR '38483-26-0':rn OR 'diacetyldichlorofluorescein'/exp OR '2, 7 dichlorofluorescein diacetate':ti,ab,kw OR '2, 7 dichlorofluorescin diacetate':ti,ab,kw OR '2, 7 difluorofluorescein':ti,ab,kw OR DCFDA:ti,ab,kw OR 'DCFH DA':ti,ab,kw OR diacetyldichlorofluorescein:ti,ab,kw OR '2044-85-1':rn

Concept 2 – EMF Exposure

'electromagnetic radiation'/de OR 'electromagnetic energ*':ti,ab,kw OR 'electromagnetic radiation':ti,ab,kw OR 'electromagnetic wave*':ti,ab,kw OR 'radiofrequency radiation'/exp OR 'Hertzian wave*':ti,ab,kw OR 'high frequency wave*':ti,ab,kw OR 'radio frequenc*':ti,ab,kw OR 'radio wave*':ti,ab,kw OR radiofrequenc*:ti,ab,kw OR radiowave*:ti,ab,kw OR 'RF electric field*':ti,ab,kw OR 'RF EMF':ti,ab,kw OR 'RF exposure*':ti,ab,kw OR 'RF field*':ti,ab,kw OR 'RF magnetic field*':ti,ab,kw OR 'RF radiation':ti,ab,kw OR 'RF wave*':ti,ab,kw OR 'short wave*':ti,ab,kw OR 'microwave radiation'/exp OR 'EHF wave*':ti,ab,kw OR 'm w field*':ti,ab,kw OR 'm w radiat*':ti,ab,kw OR 'm w range*':ti,ab,kw OR 'micro wave field*':ti,ab,kw OR 'micro wave radiat*':ti,ab,kw OR 'micro wave range*':ti,ab,kw OR 'microwave field*':ti,ab,kw OR 'microwave radiat*':ti,ab,kw OR 'microwave range*':ti,ab,kw OR 'mw field*':ti,ab,kw OR 'mw radiat*':ti,ab,kw OR 'mw range*':ti,ab,kw OR UHF:ti,ab,kw OR 'ultrahigh frequency wave*':ti,ab,kw OR 'microwave irradiation'/exp OR 'm w expos*':ti,ab,kw OR 'm w irradiat*':ti,ab,kw OR 'micro wave expos*':ti,ab,kw OR 'micro wave irradiat*':ti,ab,kw OR 'microwave expos*':ti,ab,kw OR 'microwave irradiat*':ti,ab,kw OR 'mw expos*':ti,ab,kw OR 'mw irradiat*':ti,ab,kw OR 'millimeter wave'/exp OR 'millimeter wave*':ti,ab,kw OR 'electromagnetism'/de OR 'electromagnetic environment':ti,ab,kw OR 'electromagnetic field*':ti,ab,kw OR 'electromagnetic phenomena':ti,ab,kw OR

electromagnetics:ti,ab,kw OR electromagnetism:ti,ab,kw OR radar:ti,ab,kw OR 'mobile phone'/exp OR Android:ti,ab,kw OR 'car phone*':ti,ab,kw OR 'cell phone*':ti,ab,kw OR cellphone*:ti,ab,kw OR 'cellular phone*':ti,ab,kw OR 'cellular telephone*':ti,ab,kw OR 'cordless phone*':ti,ab,kw OR iPhone*:ti,ab,kw OR 'i phone*':ti,ab,kw OR 'mobile phone*':ti,ab,kw OR 'mobile telephone*':ti,ab,kw OR 'smart phone*':ti,ab,kw OR smartphone*:ti,ab,kw OR 'cell phone use'/exp OR 'wireless communication'/exp OR 'wireless communication*':ti,ab,kw OR 'wireless technolog*':ti,ab,kw OR 'wi fi'/exp OR 'wi fi':ti,ab,kw OR wifi:ti,ab,kw OR 'specific absorption rate'/exp OR 'specific absorption rate':ti,ab,kw OR 'W/kg':ti,ab,kw OR 'digital cellular system*':ti,ab,kw OR 'global system for mobile communication'/exp OR 'global system for mobile communication*':ti,ab,kw OR 'total access communication system':ti,ab,kw OR UMTS:ti,ab,kw OR 'universal mobile telecommunication system*':ti,ab,kw OR CDMA:ti,ab,kw OR 'code division multiple access':ti,ab,kw OR WCDMA:ti,ab,kw OR 'bluetooth'/exp OR bluetooth:ti,ab,kw OR 'digital enhanced cordless telecommunication*':ti,ab,kw OR 'terrestrial trunked radio':ti,ab,kw OR WiMAX:ti,ab,kw

Concept 1 AND **Concept 2**
