# Supplementary material for: The effect of radiofrequency electromagnetic fields (RF-EMF) on biomarkers of oxidative stress in vivo and in vitro: A protocol for a systematic review
Source: Environ Int. 2022 Jan;158:106932. doi: 10.1016/j.envint.2021.106932 (PMC8668870; doi:10.1016/j.envint.2021.106932)
Supplement: Supplementary data 3 — Online appendix A3. Search strategy for Scopus. [file mmc3.pdf]

Scopus Search

Concept 1 – Oxidative Stress

INDEXTERMS("Oxidative Stress") OR TITLE-ABS-KEY("Oxidative Stress*") OR TITLE-ABS-KEY("Oxidant Stress*") OR INDEXTERMS("Protein Carbonylation") OR TITLE-ABS-KEY("Protein Carbonylation*") OR TITLE-ABS-KEY("Carbonylated Protein Formation") OR TITLE-ABS-KEY("Protein Carbonyl Formation") OR INDEXTERMS("Reactive Oxygen Species") OR TITLE-ABS-KEY("Reactive Oxygen Species") OR TITLE-ABS-KEY("Reactive Oxygen Metabolite*") OR TITLE-ABS-KEY("Active Oxygen") OR TITLE-ABS-KEY("Oxygen Radical*") OR TITLE-ABS-KEY("Pro-Oxidant*") OR INDEXTERMS("Hydroxyl Radical") OR TITLE-ABS-KEY("Hydroxyl Radical*") OR TITLE-ABS-KEY("Hydroxyl Free Radical*") OR TITLE-ABS-KEY("OH Radical*") OR CASREGNUMBER(3352-57-6) OR INDEXTERMS({Peroxides}) OR TITLE-ABS-KEY("Organic Peroxide*") OR TITLE-ABS-KEY(Peroxides) OR CASREGNUMBER(14915-07-2) OR INDEXTERMS("Hydrogen Peroxide") OR TITLE-ABS-KEY("Hydrogen Peroxide") OR TITLE-ABS-KEY(H2O2) OR TITLE-ABS-KEY("Hydrogen Dioxide") OR TITLE-ABS-KEY(Hydrogenperoxide) OR TITLE-ABS-KEY(Hydroperoxide*) OR CASREGNUMBER(7722-84-1) OR INDEXTERMS("Lipid Peroxides") OR TITLE-ABS-KEY("Lipid Peroxide*") OR TITLE-ABS-KEY(Lipoperoxide*) OR TITLE-ABS-KEY(Lipohydroperoxide*) OR TITLE-ABS-KEY("15 Hydroperoxy 5,8,11,13 Eicosatetraenoate") OR TITLE-ABS-KEY("15 Hydroperoxy 5,8,11,13 Eicosatetraenoic Acid") OR TITLE-ABS-KEY("15 Hydroperoxy 5,8,11,13 Icosatetraenoic acid") OR TITLE-ABS-KEY("15 Hydroperoxyarachidonate") OR TITLE-ABS-KEY("15 Hydroperoxyarachidonic Acid") OR TITLE-ABS-KEY("15 Hydroperoxy Arachidonic Acid") OR TITLE-ABS-KEY("15 Hydroperoxyeicosa 5,8,11,13 Tetraenoic Acid") OR TITLE-ABS-KEY("15 Hydroperoxyeicosatetraenoic Acid") OR TITLE-ABS-KEY("15 Hydroperoxy Eicosatetraenoic Acid") OR TITLE-ABS-KEY("15 Hydroperoxyicosatetraenoic Acid") OR CASREGNUMBER(67675-14-3) OR TITLE-ABS-KEY("5 HPETE") OR TITLE-ABS-KEY("5 Hydroperoxy 5,8,11,14 Eicosatetraenoic Acid") OR TITLE-ABS-KEY("5 Hydroperoxy 6,8,11,14 Eicosatetraenoate") OR TITLE-ABS-KEY("5 Hydroperoxy 6,8,11,14 Eicosatetraenoic Acid") OR TITLE-ABS-KEY("5 Hydroperoxy 6,8,11,14 Icosatetraenoic Acid") OR TITLE-ABS-KEY("5 Hydroperoxyarachidonic Acid") OR TITLE-ABS-KEY("5 Hydroperoxyeicosa 5,8,11,14 Tetraenoic Acid") OR TITLE-ABS-KEY("5 Hydroperoxyeicosa 6,8,11,14 Tetraenoic Acid") OR TITLE-ABS-KEY("5 Hydroperoxyeicosatetraenoic Acid") OR TITLE-ABS-KEY("5 Hydroperoxyicosa 6,8,11,14 Tetraenoic Acid") OR TITLE-ABS-KEY("5 Hydroperoxyicosatetraenoic Acid") OR TITLE-ABS-KEY("5 Hydroperoxy Icosatetraenoic Acid") OR CASREGNUMBER(74581-83-2) OR INDEXTERMS("Lipid Peroxidation") OR TITLE-ABS-KEY("Lipid Autooxidation*") OR TITLE-ABS-KEY("Lipid Autoxidation*") OR TITLE-ABS-KEY("Lipid Peroxidation*") OR TITLE-ABS-KEY(Lipoperoxidation) OR INDEXTERMS(Superoxides) OR TITLE-ABS-KEY(Superoxide*) OR TITLE-ABS-KEY(Superoxyde*) OR CASREGNUMBER(11062-77-4) OR INDEXTERMS("Peroxynitrous Acid") OR TITLE-ABS-KEY("Peroxynitrous Acid*") OR TITLE-ABS-KEY(Peroxynitrite*) OR TITLE-ABS-KEY(Peroxonitrite*) OR CASREGNUMBER(14691-52-2) OR INDEXTERMS("8 Hydroxy 2' Deoxyguanosine") OR TITLE-ABS-KEY("8 Hydroxy 2' Deoxyguanosine") OR TITLE-ABS-KEY("8OHdG") OR TITLE-ABS-KEY("8-Hydroxydeoxyguanosine") OR TITLE-ABS-KEY("8-Oxo-2'-Deoxyguanosine") OR TITLE-ABS-KEY("2'-Deoxy-8-Oxoguanosine") OR TITLE-ABS-KEY("8-oxodG") OR TITLE-ABS-KEY("8-oxodGuo") OR TITLE-ABS-KEY("8-oxo-dG") OR TITLE-ABS-KEY("8-OH-dG") OR TITLE-ABS-KEY("8-Oxo-Deoxyguanosine") OR TITLE-ABS-KEY("8-oxo-dGuo") OR TITLE-ABS-KEY("8-Oxo-7-Hydrodeoxyguanosine") OR TITLE-ABS-KEY("8-Oxo-7,8-Dihydrodeoxyguanosine") OR TITLE-ABS-KEY("2'-Deoxy-8-Oxo-7,8-Dihydroguanosine") OR TITLE-ABS-KEY("2'-Deoxy-7,8-Dihydro-8-Oxoguanosine") OR TITLE-ABS-KEY("7,8-Dihydro-8-Oxo-2'-Deoxyguanosine") OR TITLE-ABS-KEY("8-Oxo-7,8-Dihydro-2'-Deoxyguanosine") OR TITLE-ABS-KEY("8-Oxodeoxyguanosine") OR INDEXTERMS(Acrolein) OR TITLE-ABS-KEY(Acrolein) OR TITLE-ABS-KEY(Acroleine) OR TITLE-ABS-KEY(Acraldehyde) OR TITLE-ABS-KEY("Ethylene Aldehyde") OR TITLE-ABS-KEY("Acrylic Aldehyde") OR TITLE-ABS-KEY("Allyl Aldehyde")

OR TITLE-ABS-KEY(Propenal) OR TITLE-ABS-KEY(Acrylaldehyde) OR TITLE-ABS-KEY(Acrylylaldehyde) OR TITLE-ABS-KEY(Aqualin) OR CASREGNUMBER(107-02-8) OR INDEXTERMS("Ascorbic Acid") OR TITLE-ABS-KEY("Ascorbic Acid") OR TITLE-ABS-KEY("Cevitamic Acid") OR TITLE-ABS-KEY("Vitamin C") OR TITLE-ABS-KEY(Hybrin) OR TITLE-ABS-KEY("Potassium Ascorbate") OR TITLE-ABS-KEY("Sodium Ascorbate") OR CASREGNUMBER(134-03-2) OR CASREGNUMBER(15421-15-5) OR CASREGNUMBER(50-81-7) OR TITLE-ABS-KEY("Dehydroascorbic Acid") OR TITLE-ABS-KEY(Dehydroascorbate) OR TITLE-ABS-KEY("Dehydrovitamin C") OR CASREGNUMBER(490-83-5) OR INDEXTERMS("3-chlorotyrosine") OR TITLE-ABS-KEY("3-chlorotyrosine") OR TITLE-ABS-KEY("3-chloro-L-tyrosine") OR INDEXTERMS(Glutathione) OR TITLE-ABS-KEY(Glutathione) OR TITLE-ABS-KEY(Glutathine) OR TITLE-ABS-KEY(Glutathiol) OR TITLE-ABS-KEY(Glutathion) OR TITLE-ABS-KEY("gamma-L-Glutamyl-L-Cysteinylglycine") OR TITLE-ABS-KEY("gamma-L-Glu-L-Cys-Gly") OR TITLE-ABS-KEY("gamma Glutamylcysteinylglycine") OR TITLE-ABS-KEY("L-Glutamyl-L-Cysteinylglycine") OR TITLE-ABS-KEY(GSH) OR CASREGNUMBER(70-18-8) OR INDEXTERMS("4-hydroxy-2-nonenal") OR TITLE-ABS-KEY("4-hydroxy-2-nonenal") OR TITLE-ABS-KEY("4-hydroxynon-2-enal") OR TITLE-ABS-KEY("4-hydroxynonen-2-al") OR TITLE-ABS-KEY("4-HNE cpd") OR TITLE-ABS-KEY("4-hydroxy-2,3-nonenal") OR TITLE-ABS-KEY("4-hydroxynonenal") OR TITLE-ABS-KEY("4-hydroxy nonenal") OR CASREGNUMBER(29343-52-0) OR CASREGNUMBER(75899-68-2) OR INDEXTERMS(Isoprostanes) OR INDEXTERMS("F2-Isoprostanes") OR TITLE-ABS-KEY(Isoprostane*) OR INDEXTERMS(Dinoprost) OR TITLE-ABS-KEY(Dinoprost) OR TITLE-ABS-KEY("PG F2 alpha") OR TITLE-ABS-KEY("PGF 2 alpha") OR TITLE-ABS-KEY("PGF 2a") OR TITLE-ABS-KEY(PGF2a) OR TITLE-ABS-KEY(PGF2) OR TITLE-ABS-KEY("Prostaglandin F2alpha") OR TITLE-ABS-KEY("Prostaglandin F 2alpha") OR TITLE-ABS-KEY(PGF2alpha) OR TITLE-ABS-KEY("Prostaglandin F2") OR TITLE-ABS-KEY("Prostaglandin F 2 a") OR TITLE-ABS-KEY("Prostaglandin F 2 alpha") OR TITLE-ABS-KEY("Prostaglandin F 2a") OR TITLE-ABS-KEY("Prostaglandin F2a") OR TITLE-ABS-KEY("Prostin F 2 alpha") OR TITLE-ABS-KEY("Prostin F2 alpha") OR TITLE-ABS-KEY("U 14583") OR TITLE-ABS-KEY(U14583) OR CASREGNUMBER(551-11-1) OR INDEXTERMS(Malondialdehyde) OR TITLE-ABS-KEY(Malondialdehyde) OR TITLE-ABS-KEY("Malonic Dialdehyde") OR TITLE-ABS-KEY(Propanedial) OR TITLE-ABS-KEY(Malonyldialdehyde) OR TITLE-ABS-KEY("Malonyl Dialdehyde") OR TITLE-ABS-KEY(Malonaldehyde) OR TITLE-ABS-KEY(Malonylaldehyde) OR CASREGNUMBER(542-78-9) OR INDEXTERMS("Thiobarbituric Acid Reactive Substances") OR TITLE-ABS-KEY(TBARS) OR INDEXTERMS("Thiobarbituric Acid") OR TITLE-ABS-KEY("Thiobarbituric Acid") OR TITLE-ABS-KEY("2-Mercaptobarbituric Acid") OR TITLE-ABS-KEY(Thiobarbiturate) OR CASREGNUMBER(504-17-6) OR INDEXTERMS("Methionine Sulfoxide Reductases") OR TITLE-ABS-KEY("EC 1.8.4.5") OR TITLE-ABS-KEY("Methionine Sulfoxide Reductase*") OR TITLE-ABS-KEY("Peptide-Methionine (S)-S-oxide Reductase") OR TITLE-ABS-KEY("Selenoprotein R") OR TITLE-ABS-KEY("SelR Protein") OR TITLE-ABS-KEY("Peptide-Methionine (R)-S-oxide Reductase") OR TITLE-ABS-KEY("Methionine-R-sulfoxide Reductase*") OR TITLE-ABS-KEY("Methionine-S-oxide Reductase*") OR INDEXTERMS("3-nitrotyrosine") OR TITLE-ABS-KEY(nitrotyrosine) OR TITLE-ABS-KEY("3-mononitrotyrosine") OR TITLE-ABS-KEY("3-nitro-L-tyrosine") OR CASREGNUMBER(3604-79-3) OR INDEXTERMS("NF-E2-Related Factor 2") OR TITLE-ABS-KEY("Nrf2 protein") OR TITLE-ABS-KEY("NF-E2-related factor 2") OR TITLE-ABS-KEY("Nuclear Factor E2-Related Factor 2") OR TITLE-ABS-KEY("Nfe2l2 Protein") OR TITLE-ABS-KEY("Nuclear Factor (Erythroid-Derived 2)-Like 2 Protein") OR TITLE-ABS-KEY("nuclear factor erythroid 2-related factor 2") OR TITLE-ABS-KEY("Protein Nrf2") OR TITLE-ABS-KEY("Transcription factor NF-E2 related nuclear factor 2") OR TITLE-ABS-KEY("Transcription factor Nrf2") OR INDEXTERMS("Heme Oxygenase (Decyclizing)") OR TITLE-ABS-KEY("EC 1.14.99.3") OR TITLE-ABS-KEY("Heme Oxygenase") OR TITLE-ABS-KEY("Haem Oxygenase") OR TITLE-ABS-KEY("Hemeoxygenase-1") OR CASREGNUMBER(9059-22-7) OR TITLE-ABS-KEY(Hsp32) OR TITLE-ABS-KEY("Hsp 32") OR TITLE-ABS-KEY("HO-1 protein") OR TITLE-ABS-KEY("heat shock protein 32") OR TITLE-ABS-KEY("Hmox1 protein") OR TITLE-ABS-KEY("protein Hmox1") OR INDEXTERMS(Peroxiredoxins) OR TITLE-ABS-KEY("Alkylhydroperoxide Reductase*") OR TITLE-ABS-

KEY("EC 1.11.1.15") OR TITLE-ABS-KEY("Pag protein") OR TITLE-ABS-KEY(Peroxidoxin*) OR TITLE-ABS-KEY("Thiol-Specific Antioxidant Protein*") OR TITLE-ABS-KEY(Peroxiredoxin*) OR TITLE-ABS-KEY(PRDX3) OR TITLE-ABS-KEY("proliferation-associated protein") OR CASREGNUMBER(207137-51-7) OR INDEXTERMS(Thioredoxins) OR TITLE-ABS-KEY(Thioredoxin*) OR TITLE-ABS-KEY("Trx1 protein") OR TITLE-ABS-KEY("Trx protein") OR CASREGNUMBER(52500-60-4) OR TITLE-ABS-KEY("Txn protein") OR INDEXTERMS("Thioredoxin-Disulfide Reductase") OR TITLE-ABS-KEY("EC 1.8.1.9") OR TITLE-ABS-KEY("Trxr1 protein") OR CASREGNUMBER(9074-14-0) OR INDEXTERMS("NAD(P)H Dehydrogenase (Quinone)") OR TITLE-ABS-KEY("EC 1.6.99.2") OR TITLE-ABS-KEY("NAD(P)H dehydrogenase (quinone)") OR TITLE-ABS-KEY("diaphorase 4") OR TITLE-ABS-KEY("NAD(P)H-menadione oxidoreductase") OR TITLE-ABS-KEY("NAD(P)H: (quinone acceptor) oxidoreductase") OR TITLE-ABS-KEY("NAD(P)H quinone oxidoreductase") OR TITLE-ABS-KEY("Quinone Reductase") OR TITLE-ABS-KEY("DT Diaphorase") OR TITLE-ABS-KEY("Menadione Reductase") OR TITLE-ABS-KEY("Vitamin K Reductase") OR CASREGNUMBER(9032-20-6) OR INDEXTERMS("NADPH Dehydrogenase") OR TITLE-ABS-KEY("EC 1.6.99.1") OR TITLE-ABS-KEY("NADPH Dehydrogenase") OR TITLE-ABS-KEY("NADP Diaphorase") OR TITLE-ABS-KEY("NADPH Diaphorase") OR TITLE-ABS-KEY("Old Yellow Enzyme") OR TITLE-ABS-KEY("NADP Dehydrogenase") OR TITLE-ABS-KEY("NADPH Oxidation") OR TITLE-ABS-KEY("NADPH: (Acceptor) Oxidoreductase") OR TITLE-ABS-KEY("Nicotinamide Adenine Dinucleotide Phosphate Dehydrogenase") OR TITLE-ABS-KEY("Nicotinamide Adenine Dinucleotide Phosphate Diaphorase") OR TITLE-ABS-KEY("Triphosphopyridine Nucleotide Diaphorase") OR CASREGNUMBER(9001-68-7) OR INDEXTERMS("Glutamate-Cysteine Ligase") OR TITLE-ABS-KEY("EC 6.3.2.2") OR TITLE-ABS-KEY("Glutamate-Cysteine Ligase") OR TITLE-ABS-KEY("gamma-Glutamyl-Cysteine Synthetase") OR TITLE-ABS-KEY("Glutamylcysteine Synthetase") OR CASREGNUMBER(9023-64-7) OR INDEXTERMS(Antioxidants) OR TITLE-ABS-KEY(Antioxidant*) OR TITLE-ABS-KEY("Anti-Oxidant*") OR TITLE-ABS-KEY("Antioxidation Agent*") OR TITLE-ABS-KEY("Antioxidation Product*") OR TITLE-ABS-KEY(Antioxidative) OR TITLE-ABS-KEY(Antoxidant*) OR TITLE-ABS-KEY(Scavenger*) OR TITLE-ABS-KEY("Scavenging Agent*") OR INDEXTERMS("Antioxidant Response Elements") OR TITLE-ABS-KEY("Electrophile Response Element*") OR TITLE-ABS-KEY("EpRE binding") OR TITLE-ABS-KEY("EpRE activation") OR TITLE-ABS-KEY("EpRE induction") OR TITLE-ABS-KEY(Oxyblot*) OR INDEXTERMS("Vitamin E") OR INDEXTERMS(Tocopherols) OR TITLE-ABS-KEY(Tocoferol*) OR TITLE-ABS-KEY(Tocopherol*) OR CASREGNUMBER(1406-66-2) OR INDEXTERMS("alpha-Tocopherol") OR TITLE-ABS-KEY("Vitamin E") OR CASREGNUMBER(1406-18-4) OR CASREGNUMBER(59-02-9) OR TITLE-ABS-KEY("Alpha Tocopherolquinone") OR TITLE-ABS-KEY(Eutrophyl) OR TITLE-ABS-KEY("Tocopheryl Quinone") OR TITLE-ABS-KEY(Tocopherylquinone) OR TITLE-ABS-KEY(Tocoquinone) OR CASREGNUMBER(7559-04-8) OR INDEXTERMS(Tocotrienols) OR TITLE-ABS-KEY(Tocotrienol*) OR CASREGNUMBER(1721-51-3) OR TITLE-ABS-KEY("epc k1") OR CASREGNUMBER(127061-56-7) OR CASREGNUMBER(14101-61-2) OR INDEXTERMS("Uric Acid") OR TITLE-ABS-KEY("Uric Acid") OR TITLE-ABS-KEY("2,6,8-Trihydroxypurine") OR TITLE-ABS-KEY("2,6,8 Trioxypurine") OR TITLE-ABS-KEY(Trioxopurine) OR TITLE-ABS-KEY(Urate) OR CASREGNUMBER(69-93-2) OR INDEXTERMS(dityrosine) OR TITLE-ABS-KEY(dityrosine) OR TITLE-ABS-KEY(bityrosine) OR INDEXTERMS(dihydroethidium) OR TITLE-ABS-KEY(dihydroethidium) OR CASREGNUMBER(104821-25-2) OR TITLE-ABS-KEY("Reduced Ethidium Bromide") OR CASREGNUMBER(38483-26-0) OR INDEXTERMS(diacetyldichlorofluorescein) OR TITLE-ABS-KEY(diacetyldichlorofluorescein) OR TITLE-ABS-KEY("2',7'-dichlorofluorescin diacetate") OR TITLE-ABS-KEY("DCFH-DA") OR TITLE-ABS-KEY("2',7'-dichlorofluorescein diacetate") OR TITLE-ABS-KEY(DCFDA) OR TITLE-ABS-KEY("2',7'-difluorofluorescein") OR CASREGNUMBER(2044-85-1)

Concept 2 – EMF Exposure

INDEXTERMS("Electromagnetic Radiation") OR TITLE-ABS-KEY("Electromagnetic Wave*") OR TITLE-ABS-KEY("Electromagnetic Energ*") OR TITLE-ABS-KEY("Electromagnetic Radiation*") OR INDEXTERMS("Radio Waves") OR TITLE-ABS-KEY("Radio Wave*") OR TITLE-ABS-KEY(Radiowave*) OR TITLE-ABS-KEY("Hertzian Wave*") OR TITLE-ABS-KEY("High Frequency Wave*") OR TITLE-ABS-KEY("Short Wave*") OR TITLE-ABS-KEY("Microwave Field*") OR TITLE-ABS-KEY("Microwave Radiat*") OR TITLE-ABS-KEY("Microwave Expos*") OR TITLE-ABS-KEY("Microwave Irradiat*") OR TITLE-ABS-KEY("Microwave Range*") OR TITLE-ABS-KEY("Micro Wave Field*") OR TITLE-ABS-KEY("Micro Wave Radiat*") OR TITLE-ABS-KEY("Micro Wave Expos*") OR TITLE-ABS-KEY("Micro Wave Irradiat*") OR TITLE-ABS-KEY("Micro Wave Range*") OR TITLE-ABS-KEY("MW Field*") OR TITLE-ABS-KEY("MW Radiat*") OR TITLE-ABS-KEY("MW Expos*") OR TITLE-ABS-KEY("MW Irradiat*") OR TITLE-ABS-KEY("MW Range*") OR TITLE-ABS-KEY("M W Field*") OR TITLE-ABS-KEY("M W Radiat*") OR TITLE-ABS-KEY("M W Expos*") OR TITLE-ABS-KEY("M W Irradiat*") OR TITLE-ABS-KEY("M W Range*") OR TITLE-ABS-KEY("EHF Wave*") OR TITLE-ABS-KEY("Ultrahigh Frequency Wave*") OR TITLE-ABS-KEY(UHF) OR TITLE-ABS-KEY(Radiofrequenc*) OR TITLE-ABS-KEY("Radio Frequenc*") OR TITLE-ABS-KEY("RF Wave*") OR TITLE-ABS-KEY("RF Field*") OR TITLE-ABS-KEY("RF Electric Field*") OR TITLE-ABS-KEY("RF Magnetic Field*") OR TITLE-ABS-KEY("RF Radiation*") OR TITLE-ABS-KEY("RF Expos*") OR TITLE-ABS-KEY("RF EMF") OR TITLE-ABS-KEY("Millimeter Wave*") OR INDEXTERMS("Electromagnetic Fields") OR TITLE-ABS-KEY("Electromagnetic Environment*") OR TITLE-ABS-KEY("Electromagnetic Field*") OR TITLE-ABS-KEY("Electromagnetic Phenomen*") OR TITLE-ABS-KEY(Electromagnetics) OR TITLE-ABS-KEY(Electromagnetism) OR INDEXTERMS(Radar) OR TITLE-ABS-KEY(Radar) OR INDEXTERMS("Cell Phone") OR TITLE-ABS-KEY("Cell Phone*") OR TITLE-ABS-KEY(Cellphone*) OR TITLE-ABS-KEY("Cellular Phone*") OR TITLE-ABS-KEY("Cellular Telephone*") OR TITLE-ABS-KEY("Mobile Phone*") OR TITLE-ABS-KEY("Mobile Telephone*") OR TITLE-ABS-KEY("Cordless Phone*") OR TITLE-ABS-KEY("Car Phone*") OR INDEXTERMS(Smartphone) OR TITLE-ABS-KEY(Smartphone*) OR TITLE-ABS-KEY("Smart Phone*") OR TITLE-ABS-KEY(iPhone*) OR TITLE-ABS-KEY("i-Phone*") OR TITLE-ABS-KEY(Android) OR INDEXTERMS("Cell Phone Use") OR INDEXTERMS("Wireless Technology") OR TITLE-ABS-KEY("Wireless Technolog*") OR TITLE-ABS-KEY("Wireless Communication*") OR TITLE-ABS-KEY("Wi-Fi") OR TITLE-ABS-KEY(Wifi) OR TITLE-ABS-KEY("Specific Absorption Rate*") OR TITLE-ABS-KEY("W/kg") OR TITLE-ABS-KEY("Global System for Mobile Communication*") OR TITLE-ABS-KEY("Digital Cellular System*") OR TITLE-ABS-KEY("Universal Mobile Telecommunication System*") OR TITLE-ABS-KEY(UMTS) OR TITLE-ABS-KEY("Code Division Multiple Access") OR TITLE-ABS-KEY(CDMA) OR TITLE-ABS-KEY(WCDMA) OR TITLE-ABS-KEY(WiMAX) OR TITLE-ABS-KEY(Bluetooth) OR TITLE-ABS-KEY("Total Access Communication System") OR TITLE-ABS-KEY("Terrestrial Trunked Radio") OR TITLE-ABS-KEY("Digital Enhanced Cordless Telecommunication*")

Concept 1 AND **Concept 2**
