# Supplementary material for: The effect of radiofrequency electromagnetic fields (RF-EMF) on biomarkers of oxidative stress in vivo and in vitro: A protocol for a systematic review
Source: Environ Int. 2022 Jan;158:106932. doi: 10.1016/j.envint.2021.106932 (PMC8668870; doi:10.1016/j.envint.2021.106932)
Supplement: Supplementary data 4 — Online appendix A4. Search strategy for Web of Science Core Collection. [file mmc4.pdf]

Web of Science Search

Concept 1 – Oxidative Stress

TS=("Oxidative Stress*" OR "Oxidant Stress*" OR "Protein Carbonylation*" OR "Carbonylated Protein Formation" OR "Protein Carbonyl Formation" OR "Reactive Oxygen Species" OR "Reactive Oxygen Metabolite*" OR "Active Oxygen" OR "Oxygen Radical*" OR Pro-Oxidant* OR "Hydroxyl Radical*" OR "Hydroxyl Free Radical*" OR "OH Radical*" OR 3352-57-6 OR "Organic Peroxide*" OR Peroxides OR 14915-07-2 OR "Hydrogen Peroxide" OR H2O2 OR "Hydrogen Dioxide" OR Hydrogenperoxide OR Hydroperoxide* OR 7722-84-1 OR "Lipid Peroxide*" OR Lipoperoxide* OR Lipohydroperoxide* OR "15 Hydroperoxy 5,8,11,13 Eicosatetraenoate" OR "15 Hydroperoxy 5,8,11,13 Eicosatetraenoic Acid" OR "15 Hydroperoxy 5,8,11,13 Icosatetraenoic acid" OR "15 Hydroperoxyarachidonate" OR "15 Hydroperoxyarachidonic Acid" OR "15 Hydroperoxy Arachidonic Acid" OR "15 Hydroperoxyeicosa 5,8,11,13 Tetraenoic Acid" OR "15 Hydroperoxyeicosatetraenoic Acid" OR "15 Hydroperoxy Eicosatetraenoic Acid" OR "15 Hydroperoxyicosatetraenoic Acid" OR 67675-14-3 OR "5 HPETE" OR "5 Hydroperoxy 5,8,11,14 Eicosatetraenoic Acid" OR "5 Hydroperoxy 6,8,11,14 Eicosatetraenoate" OR "5 Hydroperoxy 6,8,11,14 Eicosatetraenoic Acid" OR "5 Hydroperoxy 6,8,11,14 Icosatetraenoic Acid" OR "5 Hydroperoxyarachidonic Acid" OR "5 Hydroperoxyeicosa 5,8,11,14 Tetraenoic Acid" OR "5 Hydroperoxyeicosa 6,8,11,14 Tetraenoic Acid" OR "5 Hydroperoxyeicosatetraenoic Acid" OR "5 Hydroperoxyicosa 6,8,11,14 Tetraenoic Acid" OR "5 Hydroperoxyicosatetraenoic Acid" OR "5 Hydroperoxy Icosatetraenoic Acid" OR 74581-83-2 OR "Lipid Autooxidation*" OR "Lipid Autoxidation*" OR "Lipid Peroxidation*" OR Lipoperoxidation OR Superoxide* OR Superoxyde* OR 11062-77-4 OR "Peroxynitrous Acid*" OR Peroxynitrite* OR Peroxonitrite* OR 14691-52-2 OR "8 Hydroxy 2' Deoxyguanosine" OR 8OHdG OR 8-Hydroxydeoxyguanosine OR 8-Oxo-2'-Deoxyguanosine OR 2'-Deoxy-8-Oxoguanosine OR 8-oxodG OR 8-oxodGuo OR 8-oxo-dG OR 8-OH-dG OR 8-Oxo-Deoxyguanosine OR 8-oxo-dGuo OR 8-Oxo-7-Hydrodeoxyguanosine OR 8-Oxo-7,8-Dihydrodeoxyguanosine OR 2'-Deoxy-8-Oxo-7,8-Dihydroguanosine OR 2'-Deoxy-7,8-Dihydro-8-Oxoguanosine OR 7,8-Dihydro-8-Oxo-2'-Deoxyguanosine OR 8-Oxo-7,8-Dihydro-2'-Deoxyguanosine OR 8-Oxodeoxyguanosine OR Acrolein OR Acroleine OR Acraldehyde OR "Ethylene Aldehyde" OR "Acrylic Aldehyde" OR "Allyl Aldehyde" OR Propenal OR Acrylaldehyde OR Acrylylaldehyde OR Aqualin OR 107-02-8 OR "Ascorbic Acid" OR "Cevitamic Acid" OR "Vitamin C" OR Hybrin OR "Potassium Ascorbate" OR "Sodium Ascorbate" OR 134-03-2 OR 15421-15-5 OR 50-81-7 OR "Dehydroascorbic Acid" OR Dehydroascorbate OR "Dehydrovitamin C" OR 490-83-5 OR 3-chlorotyrosine OR 3-chloro-L-tyrosine OR Glutathione OR Glutathine OR Glutathiol OR Glutathion OR gamma-L-Glutamyl-L-Cysteinylglycine OR gamma-L-Glu-L-Cys-Gly OR "gamma Glutamylcysteinylglycine" OR L-Glutamyl-L-Cysteinylglycine OR GSH OR 70-18-8 OR 4-hydroxy-2-nonenal OR 4-hydroxynon-2-enal OR 4-hydroxynonen-2-al OR "4-HNE cpd" OR 4-hydroxy-2,3-nonenal OR 4-hydroxynonenal OR "4-hydroxy nonenal" OR 29343-52-0 OR 75899-68-2 OR Isoprostane* OR Dinoprost OR "PG F2 alpha" OR "PGF 2 alpha" OR "PGF 2a" OR PGF2a OR PGF2 OR "Prostaglandin F2alpha" OR "Prostaglandin F 2alpha" OR PGF2alpha OR "Prostaglandin F2" OR "Prostaglandin F 2 a" OR "Prostaglandin F 2 alpha" OR "Prostaglandin F 2a" OR "Prostaglandin F2a" OR "Prostin F 2 alpha" OR "Prostin F2 alpha" OR "U 14583" OR U14583 OR 551-11-1 OR Malondialdehyde OR "Malonic Dialdehyde" OR Propanedial OR Malonyldialdehyde OR "Malonyl Dialdehyde" OR Malonaldehyde OR Malonylaldehyde OR 542-78-9 OR TBARS OR "Thiobarbituric Acid" OR "2-Mercaptobarbituric Acid" OR Thiobarbiturate OR 504-17-6 OR "Methionine Sulfoxide Reductase*" OR "EC 1.8.4.5" OR "Peptide-Methionine (S)-S-oxide Reductase" OR "Selenoprotein R" OR "SelR Protein" OR "Peptide-Methionine (R)-S-oxide Reductase" OR "Methionine-R-sulfoxide Reductase*" OR "Methionine-S-oxide Reductase*" OR nitrotyrosine OR 3-mononitrotyrosine OR 3-nitro-L-tyrosine OR 3604-79-3 OR "NF-E2-Related Factor 2" OR "Nrf2 protein" OR "Nuclear Factor E2-

Related Factor 2" OR "Nfe2l2 Protein" OR "Nuclear Factor (Erythroid-Derived 2)-Like 2 Protein" OR "nuclear factor erythroid 2-related factor 2" OR "Protein Nrf2" OR "Transcription factor NF-E2 related nuclear factor 2" OR "Transcription factor Nrf2" OR "EC 1.14.99.3" OR "Heme Oxygenase" OR "Haem Oxygenase" OR Hemeoxygenase-1 OR 9059-22-7 OR Hsp32 OR "Hsp 32" OR "HO-1 protein" OR "heat shock protein 32" OR "Hmox1 protein" OR "protein Hmox1" OR "Alkylhydroperoxide Reductase*" OR "EC 1.11.1.15" OR "Pag protein" OR Peroxidoxin* OR "Thiol-Specific Antioxidant Protein*" OR Peroxiredoxin* OR PRDX3 OR "proliferation-associated protein" OR 207137-51-7 OR Thioredoxin* OR "Trx1 protein" OR "Trx protein" OR 52500-60-4 OR "Txn protein" OR "EC 1.8.1.9" OR "Trxr1 protein" OR 9074-14-0 OR "EC 1.6.99.2" OR "NAD(P)H dehydrogenase (quinone)" OR "diaphorase 4" OR "NAD(P)H-menadione oxidoreductase" OR "NAD(P)H: (quinone acceptor) oxidoreductase" OR "NAD(P)H quinone oxidoreductase" OR "Quinone Reductase" OR "DT Diaphorase" OR "Menadione Reductase" OR "Vitamin K Reductase" OR 9032-20-6 OR "EC 1.6.99.1" OR "NADPH Dehydrogenase" OR "NADP Diaphorase" OR "NADPH Diaphorase" OR "Old Yellow Enzyme" OR "NADP Dehydrogenase" OR "NADPH Oxidation" OR "NADPH: (Acceptor) Oxidoreductase" OR "Nicotinamide Adenine Dinucleotide Phosphate Dehydrogenase" OR "Nicotinamide Adenine Dinucleotide Phosphate Diaphorase" OR "Triphosphopyridine Nucleotide Diaphorase" OR 9001-68-7 OR "EC 6.3.2.2" OR "Glutamate-Cysteine Ligase" OR "gamma-Glutamyl-Cysteine Synthetase" OR "Glutamylcysteine Synthetase" OR 9023-64-7 OR Antioxidant* OR Anti-Oxidant* OR "Antioxidation Agent*" OR "Antioxidation Product*" OR Antioxidative OR Antoxidant* OR Scavenger* OR "Scavenging Agent*" OR "Electrophile Response Element*" OR "EpRE binding" OR "EpRE activation" OR "EpRE induction" OR Oxyblot* OR Tocoferol* OR Tocopherol* OR 1406-66-2 OR "Vitamin E" OR 1406-18-4 OR 59-02-9 OR "Alpha Tocopherolquinone" OR Eutrophyl OR "Tocopheryl Quinone" OR Tocopherylquinone OR Tocoquinone OR 7559-04-8 OR Tocotrienol* OR 1721-51-3 OR "epc k1" OR 127061-56-7 OR 14101-61-2 OR "Uric Acid" OR "2,6,8-Trihydroxypurine" OR "2,6,8 Trioxypurine" OR Trioxopurine OR Urate OR 69-93-2 OR dityrosine OR bityrosine OR dihydroethidium OR 104821-25-2 OR "Reduced Ethidium Bromide" OR 38483-26-0 OR "diacetyldichlorofluorescein" OR "2',7'-dichlorofluorescin diacetate" OR DCFH-DA OR "2',7'-dichlorofluorescein diacetate" OR DCFDA OR 2',7'-difluorofluorescein OR 2044-85-1)

Concept 2 – EMF Exposure

TS=("Electromagnetic Wave*" OR "Electromagnetic Energ*" OR "Electromagnetic Radiation*" OR "Radio Wave*" OR Radiowave* OR "Hertzian Wave*" OR "High Frequency Wave*" OR "Short Wave*" OR "Microwave Field*" OR "Microwave Radiat*" OR "Microwave Expos*" OR "Microwave Irradiat*" OR "Microwave Range*" OR "Micro Wave Field*" OR "Micro Wave Radiat*" OR "Micro Wave Expos*" OR "Micro Wave Irradiat*" OR "Micro Wave Range*" OR "MW Field*" OR "MW Radiat*" OR "MW Expos*" OR "MW Irradiat*" OR "MW Range*" OR "M W Field*" OR "M W Radiat*" OR "M W Expos*" OR "M W Irradiat*" OR "M W Range*" OR "EHF Wave*" OR "Ultrahigh Frequency Wave*" OR UHF OR Radiofrequenc* OR "Radio Frequenc*" OR "RF Wave*" OR "RF Field*" OR "RF Electric Field*" OR "RF Magnetic Field*" OR "RF Radiation*" OR "RF Expos*" OR "RF EMF" OR "Millimeter Wave*" OR "Electromagnetic Environment*" OR "Electromagnetic Field*" OR "Electromagnetic Phenomen*" OR Electromagnetics OR Electromagnetism OR Radar OR "Cell Phone*" OR Cellphone* OR "Cellular Phone*" OR "Cellular Telephone*" OR "Mobile Phone*" OR "Mobile Telephone*" OR "Cordless Phone*" OR "Car Phone*" OR Smartphone* OR "Smart Phone*" OR iPhone* OR i-Phone* OR Android OR "Wireless Technolog*" OR "Wireless Communication*" OR Wi-Fi OR Wifi OR "Specific Absorption Rate*" OR "W/kg" OR "Global System for Mobile Communication*" OR "Digital Cellular System*" OR "Universal Mobile Telecommunication System*" OR UMTS OR "Code Division Multiple Access" OR CDMA OR WCDMA OR WiMAX OR Bluetooth OR "Total Access Communication System" OR "Terrestrial Trunked Radio" OR "Digital Enhanced Cordless Telecommunication*")

Concept 1 AND **Concept 2**
