# Supplementary material for: The effect of radiofrequency electromagnetic fields (RF-EMF) on biomarkers of oxidative stress in vivo and in vitro: A protocol for a systematic review
Source: Environ Int. 2022 Jan;158:106932. doi: 10.1016/j.envint.2021.106932 (PMC8668870; doi:10.1016/j.envint.2021.106932)
Supplement: Supplementary data 5 — Online appendix A5. Search strategy for the EMF-Portal. [file mmc5.pdf]

EMF-Portal Search

The search interface was set to the following parameters:

1. **Keywords** – "Or"
2. **Topics** – All topics selected
3. **Frequency ranges** – "Radio frequency (≥ 10 MHz)" and "Mobile communications"
4. **Time span** – "Complete time span"

The following search strings were entered one-at-a-time and the results downloaded in RIS format.

Search #1

"Oxidative Stress" "Oxidant Stress" "Protein Carbonylation" "Carbonylated Protein Formation" "Protein Carbonyl Formation" "Reactive Oxygen Species" "Reactive Oxygen Metabolite" "Active Oxygen" "Oxygen Radical"

Search #2

Prooxidant "Hydroxyl Radical" "Hydroxyl Free Radical" "OH Radical" "3352-57-6" "Organic Peroxide" Peroxide "14915-07-2" "Hydrogen Peroxide"

Search #3

"H2O2" "Hydrogen Dioxide" Hydrogenperoxide Hydroperoxide "7722-84-1" "Lipid Peroxide" Lipoperoxide Lipohydroperoxide "15 Hydroperoxy 5,8,11,13 Eicosatetraenoate"

Search #4

"15 Hydroperoxy 5,8,11,13 Eicosatetraenoic Acid" "15 Hydroperoxy 5,8,11,13 Icosatetraenoic acid" "15 Hydroperoxyarachidonate" "15 Hydroperoxyarachidonic Acid"

Search #5

"15 Hydroperoxy Arachidonic Acid" "15 Hydroperoxyeicosa 5,8,11,13 Tetraenoic Acid" "15 Hydroperoxyeicosatetraenoic Acid" "15 Hydroperoxy Eicosatetraenoic Acid"

Search #6

"15 Hydroperoxyicosatetraenoic Acid" "67675-14-3" "5 HPETE" "5 Hydroperoxy 5,8,11,14 Eicosatetraenoic Acid" "5 Hydroperoxy 6,8,11,14 Eicosatetraenoate"

Search #7

"5 Hydroperoxy 6,8,11,14 Eicosatetraenoic Acid" "5 Hydroperoxy 6,8,11,14 Icosatetraenoic Acid" "5 Hydroperoxyarachidonic Acid" "5 Hydroperoxyeicosa 5,8,11,14 Tetraenoic Acid"

Search #8

"5 Hydroperoxyeicosa 6,8,11,14 Tetraenoic Acid" "5 Hydroperoxyeicosatetraenoic Acid" "5 Hydroperoxyicosa 6,8,11,14 Tetraenoic Acid" "5 Hydroperoxyicosatetraenoic Acid"

Search #9

"5 Hydroperoxy Icosatetraenoic Acid" "74581-83-2" "Lipid Autooxidation" "Lipid Autoxidation" "Lipid Peroxidation" Lipoperoxidation Superoxide Superoxyde "11062-77-4"

Search #10

"Peroxynitrous Acid" Peroxynitrite Peroxonitrite "14691-52-2" "8 Hydroxy 2' Deoxyguanosine" 8OHdG "8-Hydroxydeoxyguanosine" "8-Oxo-2'-Deoxyguanosine" "2'-Deoxy-8-Oxoguanosine"

Search #11

"8-oxodG" "8-oxodGuo" "8-oxo-dG" "8-OH-dG" "8-Oxo-Deoxyguanosine" "8-oxo-dGuo" "8-Oxo-7-Hydrodeoxyguanosine" "8-Oxo-7,8-Dihydrodeoxyguanosine" "2'-Deoxy-8-Oxo-7,8-Dihydroguanosine"

Search # 12

"2'-Deoxy-7,8-Dihydro-8-Oxoguanosine" "7,8-Dihydro-8-Oxo-2'-Deoxyguanosine" "8-Oxo-7,8-Dihydro-2'-Deoxyguanosine" "8-Oxodeoxyguanosine" Acrolein Acroleine Acraldehyde "Ethylene Aldehyde" "Acrylic Aldehyde"

Search #13

"Allyl Aldehyde" Propenal Acrylaldehyde Acrylylaldehyde Aqualin "107-02-8" "Ascorbic Acid" "Cevitamic Acid" "Vitamin C"

Search #14

Hybrin "Potassium Ascorbate" "Sodium Ascorbate" "134-03-2" "15421-15-5" "50-81-7" "Dehydroascorbic Acid" Dehydroascorbate "Dehydrovitamin C"

Search #15

"490-83-5" "3-chlorotyrosine" "3-chloro-L-tyrosine" Glutathione Glutathine Glutathiol Glutathion "gamma-L-Glutamyl-L-Cysteinylglycine" "gamma-L-Glu-L-Cys-Gly"

Search #16

"gamma Glutamylcysteinylglycine" "L-Glutamyl-L-Cysteinylglycine" GSH "70-18-8" "4-hydroxy-2-nonenal" "4-hydroxynon-2-enal" "4-hydroxynonen-2-al" "4-HNE cpd" "4-hydroxy-2,3-nonenal"

Search #17

"4-hydroxynonenal" "4-hydroxy nonenal" "29343-52-0" "75899-68-2" Isoprostane Dinoprost "PG F2 alpha" "PGF 2 alpha" "PGF 2a"

Search #18

PGF2a PGF2 "Prostaglandin F2alpha" "Prostaglandin F 2alpha" PGF2alpha "Prostaglandin F2" "Prostaglandin F 2 a" "Prostaglandin F 2 alpha" "Prostaglandin F 2a"

Search #19

"Prostaglandin F2a" "Prostin F 2 alpha" "Prostin F2 alpha" "U 14583" U14583 "551-11-1" Malondialdehyde "Malonic Dialdehyde" Propanedial

Search #20

Malonyldialdehyde "Malonyl Dialdehyde" Malonaldehyde Malonylaldehyde "542-78-9" TBARS "Thiobarbituric Acid" "2-Mercaptobarbituric Acid" Thiobarbiturate

Search #21

"504-17-6" "Methionine Sulfoxide Reductase" "EC 1.8.4.5" "Peptide-Methionine (S)-S-oxide Reductase" "Selenoprotein R" "SelR Protein" "Peptide-Methionine (R)-S-oxide Reductase" "Methionine-R-sulfoxide Reductase"

Search #22

"Methionine-S-oxide Reductase" nitrotyrosine "3-mononitrotyrosine" "3-nitro-L-tyrosine" "3604-79-3" "NF-E2-Related Factor 2" "Nrf2 protein" "Nuclear Factor E2-Related Factor 2" "Nfe2l2 Protein"

Search #23

"Nuclear Factor (Erythroid-Derived 2)-Like 2 Protein" "nuclear factor erythroid 2-related factor 2" "Protein Nrf2" "Transcription factor NF-E2 related nuclear factor 2" "Transcription factor Nrf2" "EC 1.14.99.3"

Search #24

"Heme Oxygenase" "Haem Oxygenase" "Hemeoxygenase-1" "9059-22-7" Hsp32 "Hsp 32" "HO-1 protein" "heat shock protein 32" "Hmox1 protein"

Search #25

"protein Hmox1" "Alkylhydroperoxide Reductase" "EC 1.11.1.15" "Pag protein" Peroxidoxin "Thiol-Specific Antioxidant Protein" Peroxiredoxin PRDX3

Search #26

"proliferation-associated protein" "207137-51-7" Thioredoxin "Trx1 protein" "Trx protein" "52500-60-4" "Txn protein" "EC 1.8.1.9" "Trxr1 protein"

Search #27

"9074-14-0" "EC 1.6.99.2" "NAD(P)H dehydrogenase (quinone)" "diaphorase 4" "NAD(P)H-menadione oxidoreductase" "NAD(P)H: (quinone acceptor) oxidoreductase"

Search #28

"NAD(P)H quinone oxidoreductase" "Quinone Reductase" "DT Diaphorase" "Menadione Reductase" "Vitamin K Reductase" "9032-20-6" "EC 1.6.99.1"

Search #29

"NADPH Dehydrogenase" "NADP Diaphorase" "NADPH Diaphorase" "Old Yellow Enzyme" "NADP Dehydrogenase" "NADPH Oxidation" "NADPH: (Acceptor) Oxidoreductase"

Search #30

"Nicotinamide Adenine Dinucleotide Phosphate Dehydrogenase" "Nicotinamide Adenine Dinucleotide Phosphate Diaphorase" "Triphosphopyridine Nucleotide Diaphorase" "9001-68-7" "EC 6.3.2.2"

Search #31

"Glutamate-Cysteine Ligase" "gamma-Glutamyl-Cysteine Synthetase" "Glutamylcysteine Synthetase" OR "9023-64-7" Antioxidant "Anti-Oxidant" "Antioxidation Agent" "Antioxidation Product"

Search #32

Antioxidative Antoxidant Scavenger "Scavenging Agent" "Electrophile Response Element" "EpRE binding" "EpRE activation" "EpRE induction" Oxyblot

Search #33

Tocoferol Tocopherol "1406-66-2" "Vitamin E" "1406-18-4" "59-02-9" "Alpha Tocopherolquinone" Eutrophyl "Tocopheryl Quinone"

Search #34

Tocopherylquinone Tocoquinone "7559-04-8" Tocotrienol "1721-51-3" "epc k1" "127061-56-7" "14101-61-2" "Uric Acid"

Search #35

"2,6,8-Trihydroxypurine" "2,6,8 Trioxypurine" Trioxopurine Urate "69-93-2" dityrosine bityrosine dihydroethidium "104821-25-2"

Search #36

"Reduced Ethidium Bromide" "38483-26-0" "diacetyldichlorofluorescein" "2',7'-dichlorofluorescin diacetate" "DCFH-DA" "2',7'-dichlorofluorescein diacetate" DCFDA "2',7'-difluorofluorescein" "2044-85-1"
