# Supplementary material for: The effect of radiofrequency electromagnetic fields (RF-EMF) on biomarkers of oxidative stress in vivo and in vitro: A protocol for a systematic review
Source: Environ Int. 2022 Jan;158:106932. doi: 10.1016/j.envint.2021.106932 (PMC8668870; doi:10.1016/j.envint.2021.106932)
Supplement: Supplementary data 6 — Appendix B Instructions for assessment of risk of bias in individual studies. [file mmc6.docx]

APPENDIX X.

Risk of bias of the individual animal, non-randomized human and in vitro studies will be assessed using OHAT Risk of bias tool (A. A. Rooney, 2015), adapted from the Cochrane risk-of-bias tool for randomized clinical studies (Higgins & Altman, 2008). In the case of in vitro studies the tool was adapted from the OHAT tool for animal studies to in vitro models and applied to the NTP assessment of immunotoxicity associated of Perfluorooctanoic Acid (PFOA) or Perfluorooctane Sulfonate (PFOS) (A.A. Rooney, 2016).

In this tool potential sources of bias are assessed with a set of 11 questions or “domains” and an additional category to consider “other potential threats to internal validity.” Study design determines which questions [e.g., questions #1, 2, 5, 6, 7, 8, 9, 10 and 11 (or “other”) apply to experimental animal studies with a different set for case-control human studies]. Detailed criteria are provided under each question that are specific for each study design. The instructions (A. A. Rooney, 2015) outline criteria by which individual studies are assessed and define aspects of study design, conduct, and reporting that are used to assign a risk-of-bias rating for each question.

***Risk of bias domains and questions***

**Selection**

1. Was administered dose or exposure level adequately randomized?

2. Was allocation to study groups adequately concealed?

3. Did selection of study participants result in appropriate comparison groups?

**Confounding**

4. Did the study design or analysis account for important confounding and modifying variables?

**Performance**

5. Were experimental conditions identical across study groups?

6. Were the research personnel and human subjects blinded to the study group during the study?

**Attrition/Exclusion**

7. Were outcome data complete without attrition or exclusion from analysis?

**Detection**

8. Can we be confident in the exposure characterization?

9. Can we be confident in the outcome assessment?

**Selective Reporting**

10. Were all measured outcomes reported?

11. Were there no other potential threats to internal validity?

### Question Format:

- - Background
    - Definition of the general category of bias
    - Clarifying text to explain what study aspects are relevant
    - Available empirical information about the direction and magnitude of the bias
    - Information about other internal validity assessment tools that consider this element
  - Specific risk-of-bias rating instructions customized to each study type
    - Detailed criteria are outlined that define aspects of the study design, conduct, and reporting required to reach each risk-of-bias rating
    - The criteria are focused on distinguishing among the 4 risk-of-bias answers or ratings (e.g., outlining factors that separate “definitely low” from “probably low” risk of bias

### Answer Format:

***Definitely Low*** *risk of bias*:


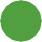


**++**

There is direct evidence of low risk-of-bias practices

(May include specific examples of relevant low risk-of-bias practices)

***Probably Low*** *risk of bias*:


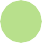


**+**

There is indirect evidence of low risk-of-bias practices OR it is deemed that deviations from low risk-of-bias practices for these criteria during the study would not appreciably bias results, including consideration of direction and magnitude of bias.

***Probably High*** *risk of bias*:


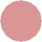

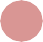


**− NR**

There is indirect evidence of high risk-of-bias practices OR there is insufficient information (e.g., not reported or “NR”) provided about relevant risk-of-bias practices

***Definitely High*** *risk of bias*:


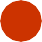


**−−**

There is direct evidence of high risk-of-bias practices

(May include specific examples of relevant high risk-of-bias practices)

***In vivo Animal Studies and in vitro cell culture studies***

1. **Was administered dose or exposure level adequately randomized?**

| **In vivo** | **In vitro** |
| --- | --- |
| **Definitely Low Risk of Bias (++)** | |
| - Direct evidence that animals were allocated to any study group including controls using a method with a random component, - **AND** direct evidence that the study used a concurrent control group as an indication that randomization covered all study groups,   **Note:** Acceptable methods of randomization include: referring to a random number table, using a computer random number generator, coin tossing, or shuffling cards (Higgins and Green 2011). | - Direct evidence that cells were allocated to any study group including controls using a method with a random component, - **AND** direct evidence that the study used a concurrent control group as an indication that randomization covered all study groups, - **OR** all cells in culture come from a homogenous cell suspension recently collected from cell culture vessels following appropriate cell culture techniques.   **Note:** Acceptable methods of randomization include: referring to a random number table, using a computer random number generator, coin tossing, or shuffling cards (Higgins and Green 2011). |
| **Probably Low Risk of Bias (+)** | |
| - Indirect evidence that animals were allocated to any study group including controls using a method with a random component (i.e., authors state random allocation, without description of the method), - **AND** evidence that the study used a concurrent control group as an indication that randomization covered all study groups, - **OR** it is deemed that allocation without a clearly random component would not appreciably bias results. | - Indirect evidence that cells were allocated to any study group including controls using a method with a random component (i.e., authors state random allocation, without description of the method), - **AND** evidence that the study used a concurrent control group as an indication that randomization covered all study groups, - **OR** it is deemed that allocation without a clearly random component would not appreciably bias results. |
| **Probably High Risk of Bias (-) or (NR)** | |
| - Indirect evidence that animals were allocated to study groups using a method with a non-random component, - **OR** indirect evidence that there was a lack of a concurrent control group, - **OR** there is insufficient information provided about how animals were allocated to study groups (record “NR” as basis for answer). | - Indirect evidence that cells were allocated to study groups using a method with a non-random component, - **OR** indirect evidence that there was a lack of a concurrent control group, - **OR** there is insufficient information provided about how animals were allocated to study groups (record “NR” as basis for answer). |
| **Definitely High Risk of Bias (--)** | |
| - Direct evidence that animals were allocated to study groups using a method with a non-random component including judgment of the investigator or the results of laboratory tests, - **OR** direct evidence that there was a lack of a concurrent control group. | - Direct evidence that cells were allocated to study groups using a method with a non-random component including judgment of the investigator or the results of laboratory tests, - **OR** direct evidence that there was a lack of a concurrent control group. |

1. **Was allocation to study groups adequately concealed?**

| **In vivo** | **In vitro** |
| --- | --- |
| **Definitely Low Risk of Bias (++)** | |
| - Direct evidence that at the time of assigning study groups the research personnel did not know what group animals were allocated to, and it is unlikely that they could have broken the blinding of allocation until after assignment was complete and irrevocable. - **Note:** Acceptable methods used to ensure allocation concealment include sequentially numbered treatment containers of identical appearance or equivalent methods. | - Direct evidence that at the time of assigning study groups the research personnel did not know what group cells were allocated to, and it is unlikely that they could have broken the blinding of allocation until after assignment was complete and irrevocable. - **Note:** Acceptable methods used to ensure allocation concealment include sequentially numbered treatment containers of identical appearance or equivalent methods. |
| **Probably Low Risk of Bias (+)** | |
| - Indirect evidence that at the time of assigning study groups the research personnel did not know what group animals were allocated to and it is unlikely that they could have broken the blinding of allocation until after assignment was complete and irrevocable, - **OR** it is deemed that lack of adequate allocation concealment would not appreciably bias results. | - Indirect evidence that at the time of assigning study groups the research personnel did not know what group cells were allocated to and it is unlikely that they could have broken the blinding of allocation until after assignment was complete and irrevocable, - **OR** it is deemed that lack of adequate allocation concealment would not appreciably bias results. This may also be the case for *in vitro* studies with very low potential differences between cell lines that comprise the different groups, e.g. animal cell lines or cells pipetted from a homogeneous cell suspension (single or mixed cell types) recently. |
| **Probably High Risk of Bias (-) or (NR)** | |
| - Indirect evidence that at the time of assigning study groups it was possible for the research personnel to know what group animals were allocated to, or it is likely that they could have broken the blinding of allocation before assignment was complete and irrevocable, - **OR** there is *insufficient* information provided about allocation to study groups (record “NR” as basis for answer). | - Indirect evidence that at *the* time of assigning study groups it was possible for the research personnel to know what group cells were allocated to, or it is likely that they could have broken the blinding of allocation before assignment was complete and irrevocable, - **OR** there is *insufficient* information provided about allocation to study groups (record “NR” as basis for answer). |
| **Definitely High Risk of Bias (--)** | |
| - Direct evidence that at *the* time of assigning study groups it was possible for the research personnel to know what group animals were allocated to, or it is likely that they could have broken the blinding of allocation before assignment was complete and irrevocable, - **OR** there is *insufficient* information provided about allocation to study groups (record “NR” as basis for answer). | - Direct evidence that at the time of assigning study groups it was possible for the research personnel to know what group cells or cell lines were allocated to, or it is likely that they could have broken the blinding of allocation before assignment was complete and irrevocable**.** |

1. **Did selection of study participants result in the appropriate comparison groups? [NA]**
2. **Did study design or analysis account for important confounding and modifying variables? [NA]**
3. **Were experimental conditions identical across study groups?**

| **In vivo** | **In vitro** |
| --- | --- |
| **Definitely Low Risk of Bias (++)** | |
| There is direct evidence that  • experimental animals were adequately sham exposed (i.e. exposure & exposure facility side effects (i.e. noise, temperature, vibrations, airflow, etc.) were consistently administered in the sham exposure control group and across treatment groups),  • **AND** non-exposure-related experimental conditions were identical across study groups (i.e., the study report explicitly provides this level of detail. | There is direct evidence that  • Samples were adequately sham exposed (i.e. exposure & exposure facility side effects (i.e. noise, temperature vibrations, airflow, etc.) were consistently administered in the sham exposure sample and across treated samples),  • **AND** non-exposure-related experimental conditions were identical across samples (i.e., the study report explicitly provides this level of detail. |
| **Probably Low Risk of Bias (+)** | |
| There is indirect evidence that  • experimental animals were sham exposed and exposure & exposure facility side effects were identical across treatment groups and in the control group,  • **OR** it is deemed that exposure & exposure facility side effects do not exist or would not appreciably bias results,  • **AND** as described above, identical non-exposure-related experimental conditions are assumed if authors did not report differences in housing or husbandry. | There is indirect evidence that  • Samples were sham exposed and exposure & exposure facility side effects were identical across treatment groups and in the control group,  • **OR** it is deemed that relevant exposure & exposure facility side effects do not exist or would not appreciably bias results,  • **AND** non-exposure-related experimental conditions were identical across samples. |
| **Probably High Risk of Bias (-) or (NR)** | |
| There is indirect evidence that  • the exposure & exposure facility generated side effects differed between control and experimental animals,  • **OR** that non-exposure-related experimental conditions were not comparable between study groups,  • **OR** there is insufficient information (e.g., not reported or “NR”) provided about relevant risk-of-bias practices. | There is indirect evidence that  • the exposure & exposure facility generated side effects differed between samples / are absent in control samples,  • **OR** the energy absorption in samples led to different experimental conditions (e.g. faster evaporation of sample liquid),  • **OR** that non-exposure-related experimental conditions were not comparable between samples,  **OR** there is insufficient information (e.g., not reported or “NR”) provided about relevant risk-of-bias practices. |
| **Definitely High Risk of Bias (--)** | |
| There is direct evidence that  • the living conditions of the control animals were different than the one of the experimental animals or differed between study groups,  • **OR** that non-exposure-related experimental conditions were not comparable between study groups. | There is direct evidence that  • the environmental conditions of the samples were not identical,  • **OR** the energy absorption in samples led to different experimental conditions (e.g. faster evaporation of sample liquid),   - **OR** that non exposure-related experimental conditions were not comparable between samples. |

1. **Were the research personnel blinded to the study group during the study?**

| **In vivo** | **In vitro** |
| --- | --- |
| **Definitely Low Risk of Bias (++)** | |
| - There is direct evidence that the research personnel were adequately blinded to study group, and it is unlikely that they could have broken the blinding during the study. Methods used to ensure blinding include central allocation; sequentially numbered treatment containers of identical appearance; sequentially numbered animal cages; or equivalent methods. | - Direct evidence that the research personnel were adequately blinded to study group, and it is unlikely that they could have broken the blinding during the study. Methods used to ensure blinding include central allocation, sequentially numbered treatment containers of identical appearance; sequentially numbered culture plates, or equivalent, - **OR** the use of robotic testing systems during the study that are deemed to eliminate the opportunity for performance bias to influence results. |
| **Probably Low Risk of Bias (+)** | |
| - There is indirect evidence that the research personnel were adequately blinded to study group, and it is unlikely that they could have broken the blinding during the study, - **OR** it is deemed that lack of adequate blinding during the study would not appreciably bias results. This would include cases where blinding was not possible but research personnel took steps to minimize potential bias, such as restricting the knowledge of study group to veterinary or supervisory personnel monitoring for overt toxicity, or randomized husbandry or handling practices (e.g., placement in the animal room, necropsy order, etc.). | - Indirect evidence that the research personnel were adequately blinded to study group, and it is unlikely that they could have broken the blinding during the study, - **OR** it is deemed that lack of adequate blinding during the study would not appreciably bias results (e.g., minimal possibility of researchers to handle animals or plates after treatment due to primarily automated procedures). |
| **Probably High Risk of Bias (-) or (NR)** | |
| - There is indirect evidence that the research personnel were not adequately blinded to study group, - **OR** there is insufficient information provided about blinding to study group during the study (record “NR” as basis for answer). | - Indirect evidence that the research personnel were not adequately blinded to study group, - **OR** there is insufficient information provided about blinding to study group during the study (record “NR” as basis for answer). |
| **Definitely High Risk of Bias (--)** | |
| - Direct evidence that the research personnel were not adequately blinded to study group. | - Direct evidence that the research personnel were not adequately blinded to study group. |

1. **Were outcome data complete without attrition or exclusion from analysis?**

| **In vivo** | **In vitro** |
| --- | --- |
| **Definitely Low Risk of Bias (++)** | |
| - There is direct evidence that loss of animals or samples was adequately addressed, and reasons were documented when animals were removed from a study. Acceptable handling of attrition includes: very little missing outcome data; reasons for missing animals unlikely to be related to outcome (or for survival data, censoring unlikely to be introducing bias); missing outcome data balanced in numbers across study groups, with similar reasons for missing data across groups; missing outcomes is not enough to impact the effect estimate, - **OR** missing data have been imputed using appropriate methods (ensuring that characteristics of data from animals are not significantly different from animals retained in the analysis). | - Direct evidence that loss of sample was adequately addressed and reasons were documented when wells or plates were removed from a study (e.g., visual observation of contamination, cells missing from wells due to pipetting error, visual morphological changes in cells unexplainable based on surrounding wells, documented removal of statistical outliers). - **Note**: Acceptable handling of attrition includes: very little missing outcome data; reasons for lost samples unlikely to be related to outcome (or for viability data, censoring unlikely to be introducing bias); missing outcome data balanced in numbers across study groups, with similar reasons for missing data across groups; missing outcomes is not enough to impact the effect. |
| **Probably Low Risk of Bias (+)** | |
| - There is indirect evidence that loss of animals or samples was adequately addressed, and reasons were documented when animals were removed from a study, - **OR** it is deemed that the proportion lost would not appreciably bias results. This would include reports of no statistical differences in characteristics of data from animals removed from the study from those remaining in the study. | - Indirect evidence that loss of samples was adequately addressed, and reasons were documented when wells or plates were removed from a study, - **OR** it is deemed that the proportion lost would not appreciably bias results. |
| **Probably High Risk of Bias (-) or (NR)** | |
| - There is indirect evidence that loss of animals or samples was unacceptably large and not adequately addressed, - **OR** there is insufficient information provided about loss of animals or samples (record “NR” as basis for answer). | - Indirect evidence that loss of samples was unacceptably large and not adequately addressed, - **OR** there is insufficient information provided about loss of samples (record “NR” as basis for answer). |
| **Definitely High Risk of Bias (--)** | |
| - There is direct evidence that loss of animals or samples was unacceptably large and not adequately addressed. Unacceptable handling of attrition or exclusion includes: reason for loss is likely to be related to true outcome, with either imbalance in numbers or reasons for loss across study groups. | - Direct evidence that loss of samples was unacceptably large and not adequately addressed. - **Note**: Unacceptable handling of attrition or exclusion includes reason for loss is likely to be related to true outcome, with either imbalance in numbers or reasons for loss across study groups. |

1. **Can we be confident in the exposure characterization?**

| **In vivo** | | **In vitro** | |
| --- | --- | --- | --- |
| **Definitely Low Risk of Bias (++)** | | | |
| Direct evidence that:   - EMF exposure was adequately characterized and monitored.   “Adequately characterized” in the above sense means that tissue internal exposure metrics have been assessed using scientifically validated methods for a representative sample of all relevant developmental stages and exposure conditions. In case of numerical computations it is a requirement that the numerical source model has been experimentally validated under operating conditions as used in the study, and that an uncertainty assessment has been performed taking into account all relevant sources of uncertainty (including at least source validation, geometries, material and tissue properties, animal orientation with respect to the incident field, shadowing effects due to neighbor animals).  “Adequately monitored” in the above sense means, that at least a valid proxy for the resulting exposure was continuously monitored throughout the experiment. For static exposure conditions (i.e., no movement of source and/or target and/or objects in the volume of RF-propagation and constant geometries) valid proxies are source RF-input power or incident field strengths at a well-defined position in the volume where the incident field can be assumed to be correlated to the exposure. In any case the correlation between the proxy and the internal exposure metric must have been obtained during characterization of exposure (see above). For non-static exposure conditions (e.g. moving animals or objects, varying geometries, etc.), monitoring of such a proxy is only acceptable, if the impact of the non-static conditions on the resulting exposure has been taken into account in the uncertainty analysis.   - **AND** whole body average SAR and possible changes thereof over time are reported (whole body SAR not required in case of explicit partial body exposure and evidence is provided or it is physically obvious that body core temperature was not affected). - **AND** organ (of interest) specific internal exposure metrics and possible changes thereof over time are reported. - **AND** an adequate dosimetric uncertainty analysis was performed suggesting that over/under-exposure is unlikely. - **AND** exposure relevant information is sufficient for reproduction of these results. | | Direct evidence that:   - EMF exposure was adequately characterized and monitored.   “Adequately characterized” in the above sense means that sample internal exposure metrics have been assessed using scientifically validated methods. In case of numerical computations it is a requirement that the numerical source model has been experimentally validated under operating conditions as used in the study, and that an uncertainty assessment has been performed taking into account all relevant sources of uncertainty (including at least source validation, geometries, material and sample properties, sample orientation and with respect to the incident field, shadowing effects due to neighbor samples).  “Adequately monitored” in the above sense means, that at least a valid proxy for the resulting exposure was continuously monitored throughout the experiment. Valid proxies are source RF-input power or incident field strengths at a well-defined position in the volume where the incident field can be assumed to be correlated to the exposure. In any case the correlation between the proxy and the internal exposure metric must have been obtained during characterization of exposure (see above).   - **AND** sample internal exposure metric is reported. - **AND** an adequate dosimetric uncertainty analysis was performed suggesting that homogeneity of exposure within the sample is sufficient as well as over/under-exposure is unlikely. - **AND** exposure relevant information is sufficient for reproduction of these results. | |
| **Probably Low Risk of Bias (+)** | | | |
| Indirect evidence that the above-mentioned criteria are met but   - Not all relevant factors that determine exposure (e.g. posture, body mass development with increasing age) have been taken into account, but no evidence for concern, - **OR** there is insufficient information provided about the validity of the exposure assessment, but no evidence for concern. | | Indirect evidence that the above-mentioned criteria are met,   - **OR** there is insufficient information provided about the validity of the exposure assessment, but no evidence for concern. | |
| **Probably High Risk of Bias (-) or (NR)** | | | |
| Indirect evidence that   - the exposure to RF-EMF, was assessed using poorly validated methods, - **OR** tissue internal exposure metrics/whole body SAR have been estimated using poorly validated assumptions but no direct evidence for concern, - **OR** tissue internal exposure metrics/whole body SAR are given but there is insufficient information provided about the exposure assessment (report N.R. as answer). - **OR** there was no sufficient exposure contrast between the exposed and reference group. Sufficient exposure contrast means that the level of exposure in “exposed” condition is higher than the exposure level of the control condition plus expanded uncertainty (CI 95%) of the exposure level assessment. This means that the uncertainty ranges of the exposure level of the exposed and the reference group do not overlap - **OR** there was a co-exposure of EMF with different frequency range (non-RF range) | | Indirect evidence that   - the exposure to RF-EMF, was assessed using poorly validated methods, - **OR** sample internal exposure metrics have been estimated using poorly validated assumptions but no direct evidence for concern, - **OR** there is insufficient information provided about the exposure assessment, but no direct evidence for concern (report N.R. as answer). - **OR** there was no sufficient exposure contrast between the exposed and reference group. Sufficient exposure contrast means that the level of exposure in “exposed” condition is higher than the exposure level of the control condition plus expanded uncertainty (CI 95%) of the exposure level assessment. This means that the uncertainty ranges of the exposure level of the exposed and the reference group do not overlap - **OR** there was a co-exposure of EMF with different frequency range (non-RF range) | |
| **Definitely High Risk of Bias (--)** | | |  |
| Direct evidence that:   - no exposure assessment of tissue internal exposure metrics or whole-body SAR has been performed, - **OR** invalid assumptions/methods have been used for exposure assessment, - **OR** the information provided is not sufficient to describe exposure. - **OR** there was no sufficient exposure contrast between the exposed and reference group. Sufficient exposure contrast means that the level of exposure in “exposed” condition is higher than the exposure level of the control condition plus expanded uncertainty (CI 95%) of the exposure level assessment. This means that the uncertainty ranges of the exposure level of the exposed and the reference group do not overlap. - **OR** there was a co-exposure of EMF with different frequency range (non-RF range) | Direct evidence that:   - no exposure assessment of sample internal exposure metrics has been performed, - **OR** invalid assumptions/methods have been used for exposure assessment, - **OR** the information provided is not sufficient to describe exposure. - **OR** there was no sufficient exposure contrast between the exposed and reference group. Sufficient exposure contrast means that the level of exposure in “exposed” condition is higher than the exposure level of the control condition plus expanded uncertainty (CI 95%) of the exposure level assessment. This means that the uncertainty ranges of the exposure level of the exposed and the reference group do not overlap. - **OR** there was a co-exposure of EMF with different frequency range (non-RF range**)** | |  |

1. **Can we be confident in the outcome assessment?**

| **In vivo** | **In vitro** |
| --- | --- |
| **Definitely Low Risk of Bias (++)** | |
| - There is direct evidence that the outcome was assessed using well-established methods and markers.   - **AND** there is direct evidence that the outcome assessors were blinded to the study group, and it is unlikely that they could have broken the blinding prior to reporting outcomes.   - **AND** individual data properly reported (e.g. not just means, standard deviation, confidence intervals).   - **AND** the sensitivity of the detection of biomarkers was reported.   - **AND i**nformation relevant for the experimental set-up is sufficient for reproduction of the results.   - **AND** an internal standard was included for the methods used to detect biomarkers of oxidative stress.   - **AND** characterized the sample population by e.g. sex, age, weight and health status (for example diabetes). | - There is direct evidence that the outcome was assessed using well-established methods and markers. - **AND** there is direct evidence that the outcome assessors were blinded to the study group, and it is unlikely that they could have broken the blinding prior to reporting outcomes. - **AND** individual data properly reported (e.g. not just means, standard deviation, confidence intervals). - **AND** the sensitivity of the detection of biomarkers was reported. - **AND** information relevant for the experimental set-up is sufficient for reproduction of the results. - **AND** an internal standard was included for the methods used to detect biomarkers of oxidative stress. - **AND** characterized the sample population by e.g. sex, age, weight and health status (for example diabetes). |
| **Probably Low Risk of Bias (+)** | |
| - There is indirect evidence that the outcome was assessed using acceptable methods. | - There is indirect evidence that the outcome was assessed using acceptable methods. |
| **Probably High Risk of Bias (-) or (NR)** | |
| - - There is indirect evidence that the outcome assessment method is an insensitive instrument),   - **OR** the length of follow up differed among study groups,   - **OR** there is indirect evidence that it was possible for outcome assessors to infer the study group prior to reporting outcomes,   - **OR** no internal standard was included,   - **OR** the sample population was not characterized. | - There is indirect evidence that the outcome assessment method is an insensitive instrument, - **OR** the length of follow up differed among study groups, - **OR** there is indirect evidence that it was possible for outcome assessors to infer the study group prior to reporting outcomes, - **OR** no internal standard was included, - **OR** the sample population was not characterized. |
| - **Definitely High Risk of Bias (--)** | |
| - There is direct evidence that the outcome assessment method is an insensitive instrument,   - **OR** there is direct evidence that length of follow up differs among study groups,   - **OR** the data reporting is inadequate (i.e. with no standard deviation or standard error),   - **OR** no information provided on the limit of detection for a biomarker when values are reported as “undetectable” in at least one study group. - **OR** not sufficient information on the experimental set-up is given to reproduce the results, - **OR** no internal standard was included**.** - **OR** the sample population was not characterized. | - There is direct evidence that the outcome assessment method is an insensitive instrument,   - **OR** there is direct evidence that length of follow up differs among study groups,   - **OR** the data reporting is inadequate (i.e. with no standard deviation or standard error),   - **OR** no information provided on the limit of detection for a biomarker when values are reported as “undetectable” in at least one study group. - **OR** not sufficient information on the experimental set-up is given to reproduce the results, - **OR** no internal standard was included, - **OR** the sample population was not characterized. |

1. **Were all measured outcomes reported?**

| **In vivo** | **In vitro** |
| --- | --- |
| **Definitely Low Risk of Bias (++)** | |
| - Direct evidence that   - All outcomes (primary and secondary) outlined in the protocol, methods, abstract, and/or introduction (that are relevant for the evaluation) have been reported. This would include outcomes reported with sufficient detail to be included in meta-analysis or fully tabulated during data extraction and analyses had been planned in advance. | - Direct evidence that   - All of outcomes (primary and secondary) outlined in the protocol, methods, abstract, and/or introduction (that are relevant for the evaluation) have been reported. This would include outcomes reported with sufficient detail to be included in meta-analysis or fully tabulated during data extraction and analyses had been planned in advance. |
| **Probably Low Risk of Bias (+)** | |
| - Indirect evidence that   all outcomes (primary and secondary) outlined in the protocol, methods, abstract, and/or introduction (that are relevant for the evaluation) have been reported,   - **OR** analyses that had not been planned in advance (i.e., retrospective unplanned subgroup analyses) are clearly indicated as such and deemed that unplanned analyses were appropriate and selective reporting would not appreciably bias results (e.g., appropriate analyses of an unexpected effect). | - Indirect evidence that   all outcomes (primary and secondary) outlined in the protocol, methods, abstract, and/or introduction (that are relevant for the evaluation) have been reported,   - **OR** analyses that had not been planned in advance (i.e., retrospective unplanned subgroup analyses) are clearly indicated as such and deemed that unplanned analyses were appropriate and selective reporting would not appreciably bias results (e.g., appropriate analyses of an unexpected effect). |
| **Probably High Risk of Bias (-) or (NR)** | |
| - Indirect evidence that   - NOT all measured outcomes (primary and secondary) outlined in the protocol, methods, abstract, and/or introduction (that are relevant for the evaluation) have been reported,      - - **OR** there is insufficient information provided about selective outcome reporting (record “NR” as basis for answer). - **OR** outcomes were reported with insufficient detail such as only reporting that results were statistically significant (or not). | - Indirect evidence that   - NOT all measured outcomes (primary and secondary) outlined in the protocol, methods, abstract, and/or introduction (that are relevant for the evaluation) have been reported,   - **OR** there is insufficient information provided about selective outcome reporting (record “NR” as basis for answer). - **OR** outcomes were reported with insufficient detail such as only reporting that results were statistically significant (or not). |
| **Definitely High Risk of Bias (--)** | |
| - Direct evidence that - Measured outcomes outlined in the protocol, methods, abstract, and/or introduction (that are relevant for the evaluation) have not been reported. | - Direct evidence that - Measured outcomes outlined in the protocol, methods, abstract, and/or introduction (that are relevant for the evaluation) have not been reported. |

1. **Were there no other potential threats to internal validity? (Exposure specific: exposure-induced temperature effects)**

This question will be used to examine individual studies for appropriate statistical methods (e.g., confirmation of homogeneity of variance for ANOVA and other statistical tests that require normally distributed data). It will also be used for risk- of-bias considerations that do not fit under the other questions.

1. **Were there no other potential threats to internal validity? (Exposure specific: exposure-induced temperature effects)**

This question will be used to examine individual studies for appropriate statistical methods (e.g., confirmation of homogeneity of variance for ANOVA and other statistical tests that require normally distributed data). It will also be used for risk- of-bias considerations that do not fit under the other questions.

| **In vivo** | **In vitro** |
| --- | --- |
| **Definitely Low Risk of Bias (++)** | |
| Direct evidence that:   - An adequate temperature analysis of body core temperature during exposure has been performed for a representative sample of all relevant developmental stages and exposure conditions.   Adequate in the above sense include techniques that are representing the body core temperatures and are not prone to errors due to local temperature gradients (e.g. close to the skin). CW exposure: temperature needs to be obtained in the steady state. Intermittent exposure: time profiles of the temperature during the exposure pattern need to be obtained. An uncertainty analysis is also required.   - **AND** in case of local exposure an adequate temperature analysis of local temperature during exposure has been performed.   Adequate in the above sense include either calibrated measurements inside the tissue with sufficient small probes that do not affect EMF absorption/distribution or experimentally validated computer simulations. CW exposure: temperature needs to be obtained in the steady state. Intermittent exposure: time profiles of the temperature during the exposure pattern need to be obtained. An uncertainty analysis is also required.   - **OR** there is insufficient information provided about EMF exposure induced temperature alterations, but according to the provided exposure data no evidence for any biologically or outcome-relevant exposure-induced temperature alteration. | Direct evidence that:   - An adequate analysis of the Exposure-induced temperature distribution in the sample volume has been performed.   Adequate in the above sense include either calibrated spatially resolved measurements with sufficient small probes that do not affect EMF absorption/distribution Or experimentally validated computer simulations. CW exposure: temperature needs to be obtained in the steady state. Intermittent exposure: time profiles of the temperature during the exposure pattern need to be obtained. An uncertainty analysis is also required.   - **OR** there is insufficient information provided about EMF exposure induced temperature elevations, but according to the provided exposure data no evidence for any biologically or outcome- relevant exposure-induced temperature alteration in the sample. |
| **Probably Low Risk of Bias (+)** | |
| Indirect evidence that   - A temperature analysis in the above sense has been performed, - **OR** there is insufficient information provided about EMF exposure induced temperature alterations, but according to the provided exposure data no evidence for any biologically or outcome- relevant exposure-induced temperature alteration | Indirect evidence that   - A temperature analysis in the above sense has been performed, - **OR** there is insufficient information provided about EMF exposure induced temperature alterations, but according to the provided exposure data no evidence for any biologically or outcome- relevant exposure-induced temperature alteration in the sample. |
| **Probably High Risk of Bias (-) or (NR)** | |
| Indirect evidence that   - EMF-induced temperature elevations have not been adequately assessed, - **AND** the intensity of exposure is in a range where relevant exposure-induced temperature elevations cannot be ruled out. | Indirect evidence that   - EMF-induced temperature elevations have not been adequately assessed, - **AND** the intensity of exposure is in a range where relevant exposure-induced temperature elevations cannot be ruled out. |
| **Definitely High Risk of Bias (--)** | |
| Direct evidence that:   - EMF exposure induced temperature elevations have not been adequately assessed. - **AND** the intensity of exposure is in a range where relevant exposure-induced temperature elevations cannot be ruled out. c | Direct evidence that:   - EMF exposure induced temperature elevations have not been adequately assessed. - **AND** the intensity of exposure is in a range where relevant exposure-induced temperature elevations cannot be ruled out. |

**References.**

Higgins, J. P., & Altman, D. G. (2008). Assessing Risk of Bias in Included Studies. In *Cochrane Handbook for Systematic Reviews of Interventions: Cochrane Book Series*. https://doi.org/10.1002/9780470712184.ch8

Higgins JPT, Green S, eds. 2011. Cochrane Handbook for Systematic Reviews of Interventions. Version 5.1.0 (updated March 2011). Available: <http://handbook.cochrane.org/>

Rooney, A. (2016). NTP Monograph Immunotoxicity Associated with Exposure to Perfluorooctanoic Acid (PFOA) or Perfluorooctane Sulfonate (PFOS). *National Toxicology Program*.

Rooney, A. A. (2015). OHAT risk-of-bias tool. Retrieved from https://ntp.niehs.nih.gov/ntp/ohat/pubs/riskofbiastool_508.pdf
