# Supplementary material for: The effect of radiofrequency electromagnetic fields (RF-EMF) on biomarkers of oxidative stress in vivo and in vitro: A protocol for a systematic review
Source: Environ Int. 2022 Jan;158:106932. doi: 10.1016/j.envint.2021.106932 (PMC8668870; doi:10.1016/j.envint.2021.106932)
Supplement: Supplementary data 8 — Online appendix C2 GRADE tool for animal studies. [file mmc8.docx]

**Certainty^[[1]](#footnote-1)^* of the evidence for associations of animal studies between RF EMF exposure and adverse health outcomes**

GRADE has been developed to standardize the approach to judging the certainty of the effects of interventions. The idea is that the comparability of the judgements increases when all assessors look at the same arguments underpinning their certainty. The GRADE framework assumes that the perfect evidence yields high certainty in the conclusions of a systematic review. Judgements are made of five domains that would decrease the certainty in the evidence to very low. For non-experimental studies another three domains that would increase the certainty in the evidence. The factors have been elaborated in a series of articles in the Journal of Clinical Epidemiology.^1^

To assess if there is an effect on health of RF EMF, we will use experimental studies, human observational studies, animal studies and in vitro studies. The GRADE approach has to be elaborated to a lesser or greater extent for study designs that are different from experimental studies.

For observational studies of exposure, most of the reasoning used by GRADE can be equally well be used. However, at some points there is a need for elaboration or clarification on how to use the GRADE criteria for observational studies of exposure.^2^ For example, for the downgrading factor consistency, elaboration is needed because heterogeneity in observational studies is much larger than in randomized experiments.

The same holds for animal studies of environmental exposure which are usually experimental studies in which animals are at random exposed, non-exposed (sham) or exposed to a lower level. Part of the reasoning is the same as for human experiments such as decreasing the certainty of the evidence due to limitations in the study design such as lack of randomisation. On the other hand, the judgement must be adapted because the effects of the exposure can be different in animals compared to humans. This is assessed for the domain of directness that has to be elaborated for animal studies. Here, I will follow and elaborate the adaptations for animal studies proposed by the GRADE working group.^3^

It is good to note that the judgement of the certainty of the evidence is about the conclusions of the systematic review and that there are no specific requirements needed for the review to be able to apply GRADE. Preferably, the results of the review are presented in the form of a pooled effect-size such as a relative risk (RR) resulting from a meta-analysis of several studies. Usually, then there is still a conclusion of the systematic review about there being an effect of the exposure or not but this is then presented in a narrative way. Also, for this type of narrative synthesis we would like to judge the certainty and this can also be done using GRADE.^4^ Also, if the conclusion of the review is based only on one study, GRADE can be applied. The domains that require more studies such as inconsistency and publication bias are then not applicable. If there is only one study, then there will usually be reasons to downgrade for imprecision with more levels unless this is the mega-RCT that delivered definite results.

Behind each down and upgrading factor there is an idea why this factor is important to consider and what would be good reasons for downgrading or not downgrading. These ideas are well explained in the series in the Journal of Clinical Epidemiology.^1^ When making elaborations or adaptations it is good to build on these ideas.

Our systematic review investigates effects of exposure on multiple biomarkers and therefore we will judge the certainty of evidence across all biomarkers in all studies. The assessment of the evidence across all studies will be based on these endpoints (e.g. biomarkers of oxidative stress).

It is suggested to start the rating of the certainty of the evidence for animal studies at high certainty evidence just like in human experimental studies. Then, depending on which criteria for which domains are met, downgrade the certainty of the evidence. Animal studies are experiments and very different from human epidemiological studies. The upgrading domains used for the human observational studies do not apply to the animal studies. Below there is an elaboration of the criteria to downgrade the certainty of the evidence for animal studies of exposure to RF EMF.

1. ***Reasons for downgrading***
2. *Limitations in studies: rate down with one, two or three levels*

The GRADE domain limitations in studies can lead to downgrading with one, two or three levels if the studies that form the evidence base for the specific association are at serious, very serious or even more serious risk of bias. A judgement should be based on the number of studies and the impact they have in the meta-analysis, as well as the seriousness of the risk of bias in these studies. One small study with very serious risk of bias but hardly an influence on the meta-analysis should not be a reason to downgrade, but two big studies with a considerable weight in the meta-analysis should. If the sensitivity analysis for risk of bias shows a considerable effect on the effect-size, the conclusions could be based on the high-quality studies only. In that case, there is no reason to downgrade because the body of evidence on which the conclusions are based is considered at low risk of bias only.^5^

1. *Indirectness: rate down with one or two levels*

For the domain of indirectness, one should consider how well the PECO in the studies in the systematic review reflects the original PECO that was formulated at the start of the systematic review process. For animal studies, this is a two-step process: first assess how well the animal PECO has been addressed and then how well the study addresses the human PECO.

*2a. Assess directness of the animal PECO*

Any element of the PECO can be assessed as only indirectly answering that PECO. For example, the use of only very young or special animals makes it difficult to generalize to the animal species in general. The use of extreme local exposures would make it difficult to generalize to low-level whole-body exposure. The use of a surrogate outcome such as hormone level changes instead of fertility outcomes makes it difficult to generalize to fertility in animals.

If there are considerable differences between the elements of the PECO, then rate down the body of evidence with one or two levels.

*2b. Assess the directness of the evidence for the human PECO*

The directness to the human PECO depends on how well the animal model can be translated to the human situation. Based on biological differences animal models can be more or less comparable to what happens in humans. In the appendix there is a list of translational factors that can be applied to see if how directly the animal evidence answers the human PECO.

If there is a large difference rate down the body of evidence with one or two levels.

*Provide in the end two different GRADE ratings: one with the directness assessed for the animal PECO and one with the directness assessed for the human PECO.*

1. *Inconsistency: rate down with one or two levels*

Inconsistency between studies means that there is a considerable difference in effect size between studies. For example, if there are studies in the body of evidence that show a preventive effect and also studies that show a harmful effect, this indicates inconsistency or serious heterogeneity. Usually there is more heterogeneity in animal studies than in human experimental studies because the animal studies are more of an exploratory character or the exposure can have different effects in different species. Therefore, it is important to try to find out if heterogeneity in effect is due to specific differences between studies. This is the objective of a subgroup analysis comparing the effects for example in studies with rats with those in studies with rabbits. If the effects are consistent within a species but different across species and heterogeneity can be explained, there is no reason for concern and no need to down grade. However, it is important to note that in this case the results should be reported for the subgroups and not for the whole group. Inconsistency can always be judged, also when a meta-analysis is not possible. Underneath, first the situation will be explained where meta-analysis is possible and then when this is not possible

*3a. Inconsistency when meta-analysis is possible*

Heterogeneity can be measured statistically by means of the I^2^ statistic which varies between 0 and 100%, with 0% indicating no heterogeneity and 100% large heterogeneity. Because the I^2^ statistic is a relative measure it is difficult to make a judgement of the absolute amount of heterogeneity. Therefore, it is strongly advocated to use the prediction interval estimated from the underlying distribution of effect estimates.^6-8^ The prediction interval provides an estimate of the distribution of the true effect sizes. For an 80% prediction interval the true effect size for 80% of all studies falls in this interval. The reason to take an 80% confidence interval and not a 95% PI is that the 80% PI provides a more conservative approach in making a judgement. This tells us if the effect is consistent across studies or if it varies substantially. It also tells us if the effect is harmful in all populations, or beneficial in some and harmful in others.

To make a judgement about the amount of heterogeneity that would be a reason for concern and a reason to downgrade if it cannot be explained the following approach can be followed. If the 80% PI overlaps with the null value (RR=1) it means that studies show both beneficial and harmful effects of exposure. If the 80% prediction interval for a specific meta-analysis of RRs is of the same size as the 95% confidence interval of the pooled effect estimate it indicates that there is no more variation in effect sizes than the statistical uncertainty. Then there is no reason for concern about heterogeneity. However, if the prediction interval is considerably wider than the confidence interval (for example double the size) and overlaps with 1, then there is reason for concern about heterogeneity. The effect sizes of the studies vary so much that with different samples of studies the conclusions of the meta-analysis could be substantially different apart from statistical uncertainty. In this case, downgrade the certainty of the body of evidence with one level.

*3b Inconsistency when meta-analysis is not possible*

Basically, the same approach as above should be followed. First, judge the direction of the effects in the studies that are used to draw a conclusion: is it harmful or beneficial or is there no effect. If the direction is the same in the majority of the studies (75%), including the bigger studies, there is no reason for concern. If it is less, then assess the effect in subgroups of studies and see if this increases consistency within subgroups. If it does report results per subgroup and do not downgrade. If consistency does not increase in subgroups, rate down with one or two levels.

1. *Imprecision rate down with one or two levels*

Precision of the pooled effect size is another domain to judge for downgrading. If there are only a few participants and the confidence interval around the pooled effect-size is wide, we are less inclined to believe that the results reflect the true results. Ideally, this should be based on the optimal information size but this is difficult to assess for animal studies. Therefore, it is more practical to base it on the sample size of a sufficiently powered trial. As proposed by Cohen, 400 participants would be a sufficient sample size to detect a small effect of standard mean difference (SMD)=0.2.^9^ However, at this time, no threshold for any of these parameters concerning a biological outcome has been established.

If you have a pooled effect size, first, consider whether the boundaries of the confidence interval (CI) are on the same side of the null value. If they are, and the sample in the systematic review meets the optimal information size or the sample size is at least 400, do not rate down.

If the CI crosses the null-value and there is both the possibility of an appreciable (25%) benefit or an appreciable (25%) harm rate down for imprecision irrespective of where the point estimate and CI lie. If the sample size is smaller than 400, rate down with one level.

1. *Publication bias: rate down with one level*

Publication bias will be assessed by judging the funnel plot and if there is, a reason to assume that publication bias is likely, also by the Egger’s test. If the funnel plot, upon visual inspection shows that small studies with non-harmful effects are missing, this would be an indication of publication bias. We will then use Egger’s test to confirm this. Since the test is not very powerful, this is a conservative estimate of publication bias. In addition, the test does not need any assumptions. If there is less than ten studies per exposure-outcome combination, this cannot be reliably assessed.

**References**

1. Guyatt G, Oxman AD, Akl EA, et al. GRADE guidelines: 1. Introduction-GRADE evidence profiles and summary of findings tables. *J Clin Epidemiol* 2011;64(4):383-94. doi: 10.1016/j.jclinepi.2010.04.026 [published Online First: 2011/01/05]

2. Morgan RL, Thayer KA, Bero L, et al. GRADE: Assessing the quality of evidence in environmental and occupational health. *Environ Int* 2016;92-93:611-6. doi: 10.1016/j.envint.2016.01.004 [published Online First: 2016/02/02]

3. Hooijmans CR, de Vries RBM, Ritskes-Hoitinga M, et al. Facilitating healthcare decisions by assessing the certainty in the evidence from preclinical animal studies. *PLoS One* 2018;13(1):e0187271. doi: 10.1371/journal.pone.0187271 [published Online First: 2018/01/13]

4. Murad MH, Mustafa RA, Schunemann HJ, et al. Rating the certainty in evidence in the absence of a single estimate of effect. *Evid Based Med* 2017;22(3):85-87. doi: 10.1136/ebmed-2017-110668 [published Online First: 2017/03/23]

5. Guyatt GH, Oxman AD, Vist G, et al. GRADE guidelines: 4. Rating the quality of evidence--study limitations (risk of bias). *J Clin Epidemiol* 2011;64(4):407-15. doi: 10.1016/j.jclinepi.2010.07.017 [published Online First: 2011/01/21]

6. Borenstein M. Common mistakes in meta-analysis and how to avoid them. New Jersey USA: Biostat Inc 2019.

7. Borenstein M, Higgins JP, Hedges LV, et al. Basics of meta-analysis: I(2) is not an absolute measure of heterogeneity. *Res Synth Methods* 2017;8(1):5-18. doi: 10.1002/jrsm.1230 [published Online First: 2017/01/07]

8. IntHout J, Ioannidis JP, Rovers MM, et al. Plea for routinely presenting prediction intervals in meta-analysis. *BMJ Open* 2016;6(7):e010247. doi: 10.1136/bmjopen-2015-010247 [published Online First: 2016/07/14]

9. Norman G, Monteiro S, Salama S. Sample size calculations: should the emperor's clothes be off the peg or made to measure? *BMJ* 2012;345:e5278. doi: 10.1136/bmj.e5278 [published Online First: 2012/08/25]

Appendix from Hooimans et al ^3^

S2 File: Examples of assessing indirectness of evidence from preclinical animal studies

The population or the induced disease in the studies differs substantially from the population or the disease in which we are interested

- Matching preclinical conditions to clinical setting (also with regard to physiological derangement; i.e. should ideally be similar)

- Assessment of multiple manifestations of disease phenotype

- Species: multiple species tested; comparable results between different species

- Animal model/disease: similarity of disease in clinical setting, e.g. how is disease induced in the animals

- Model match to human manifestation of disease, interventions and sex, age and co-morbidities of patients in clinical setting

- Characterization of animal properties at baseline

- Co-morbidities

The Intervention of the studies differs substantially from intervention in which we are interested

- Optimization of complex treatment parameters

- Matching timing of treatment delivery to clinical setting

- Matching route/method of treatment delivery to clinical setting

- Definition of treatment

- Faithful delivery of intended treatment

- Theoretical relationship between experimental operations/interventions and clinical scenario

- Treatment response along mechanistic pathway

- Use of validated assay for molecular pathways assessment

- Treatment interactions with clinically relevant co-morbidities

The Comparison of the studies differs substantially from comparison in which we are interested

- Appropriate control group

- Comparability of control group characteristics to those of previous studies

- Indirect comparisons

The Outcome of the studies differs substantially from outcome in which we are interested

- Degree of characterization and validity of outcome measure chosen

- Assessment of outcome at late/clinically-relevant time points

1. *The GRADE working group uses currently the term certainty of the evidence. Others use ‘quality’ of the evidence or the ‘confidence’ in the evidence. These are all considered synonyms indicating the same concept. [↑](#footnote-ref-1)
